# Supplementary figures and images for: Genetic factors contributing to extensive variability of sex-specific hepatic gene expression in Diversity Outbred mice
Source: PLoS One. 2020 Dec 2;15(12):e0242665. doi: 10.1371/journal.pone.0242665 (PMC7710091; doi:10.1371/journal.pone.0242665)

## Slide 1
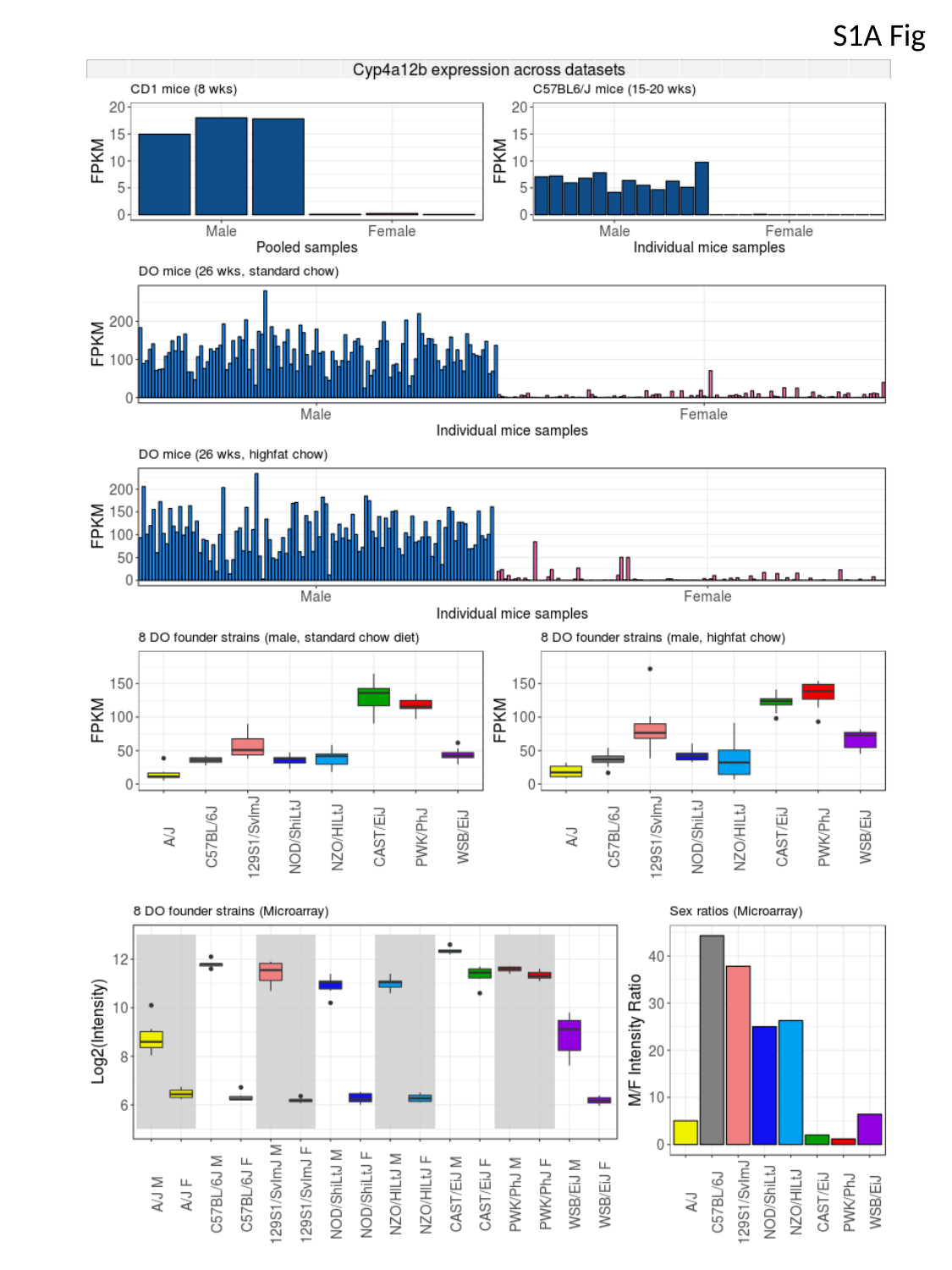

S1A Fig

## Slide 2
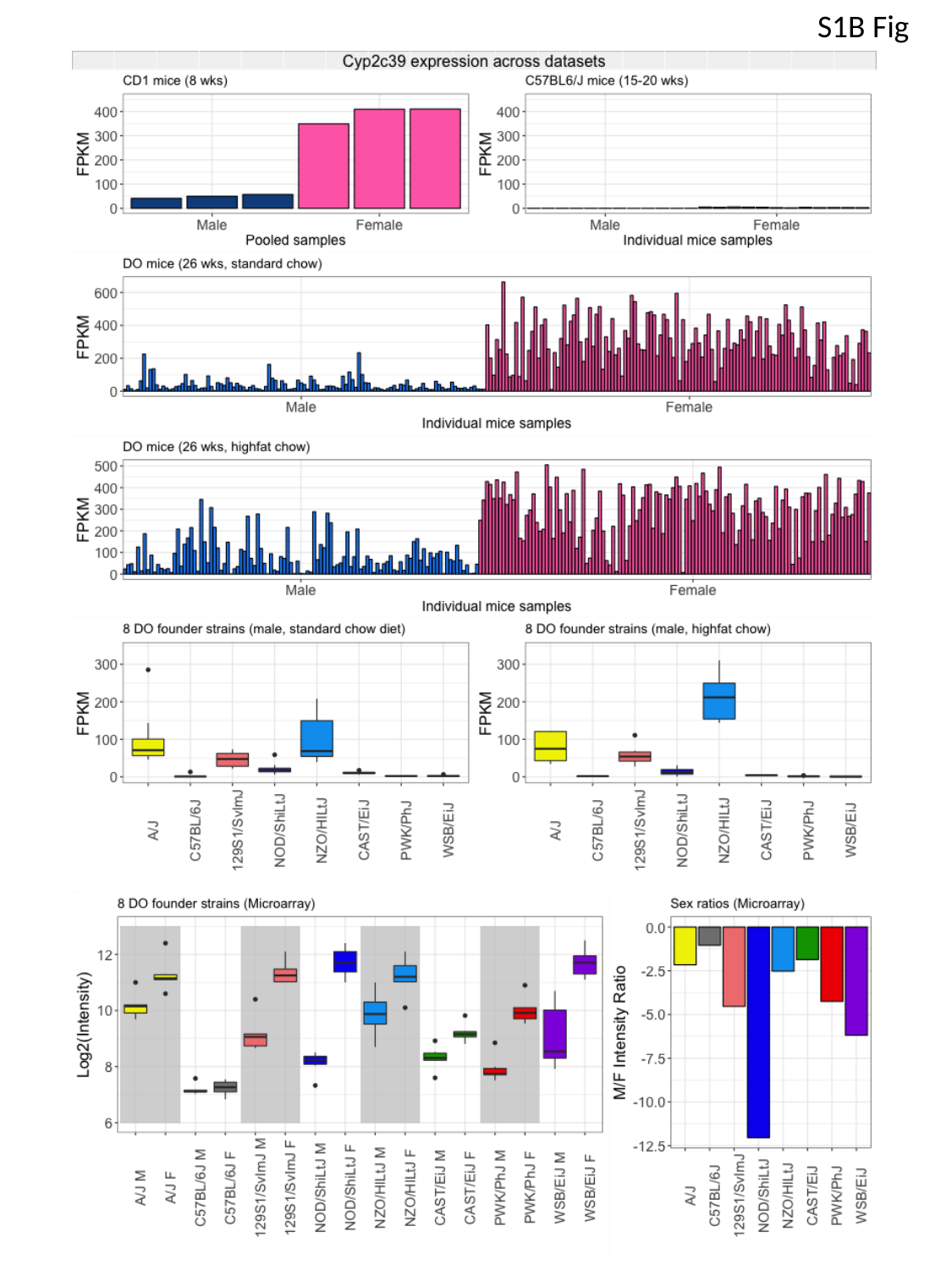

S1B Fig

Supplement: S1 Fig — Gene expression in individual mouse livers across strains: male and female CD-1 mice (first row; left), male and female C57BL/6J mice (first row, right), male and female DO mice fed a standard chow diet (second row), or fed a high fat diet (third row). The fourth row shows box plots of gene expression level (FPKM) based on 128 individual male livers for DO founder mice fed a standard chow diet (left), or fed a high fat diet (right), and for male and female DO founder strain mice (fifth row; left). The first four rows present gene expression values determined by RNA-seq (FPKM values), while the gene expression on the fifth row was determined by microarray analysis. Male/Female expression ratios across the DO founder strains based on the microarray dataset are also presented (fifth row; right). Examples shown are for Cyp4a12b, a male-specific gene (A), where male-biased expression is reduced or lost in PWK/PhJ mice, and for Cyp2c39, a female-specific gene (B), where female-biased expression is lost in C57BL/6J mice. (PPTX) [file pone.0242665.s001.pptx]

## Slide 1
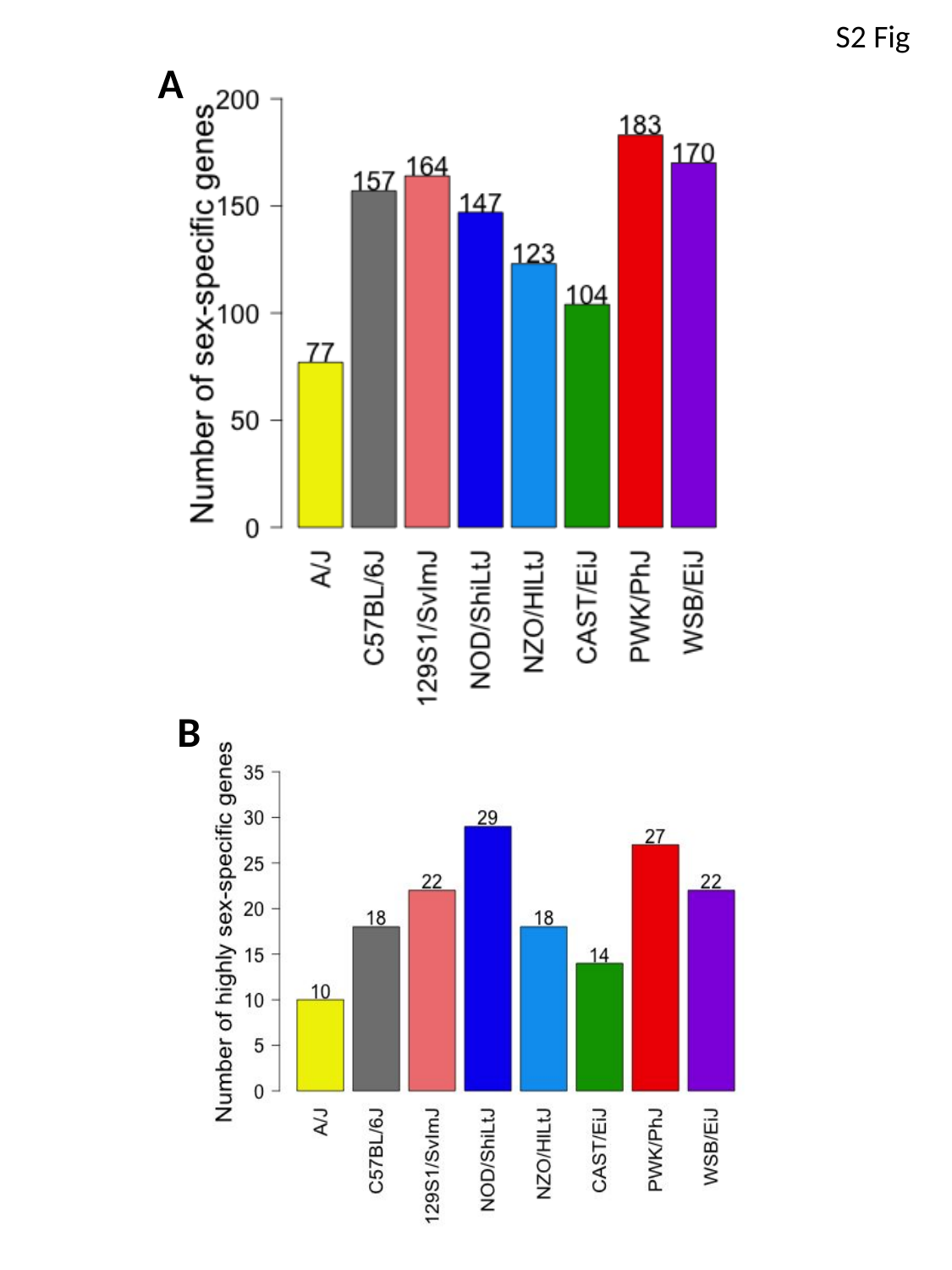

S2 Fig
A
B

Supplement: S2 Fig — Shown are the number of sex-specific protein-coding genes (A) or number of highly sex-specific protein-coding genes (B) (male/female |fold-change| > 4 at FDR < 0.05) in at least one DO mouse founder strain based on the microarray dataset) for each founder mouse strain. (PPTX) [file pone.0242665.s002.pptx]

## Slide 1
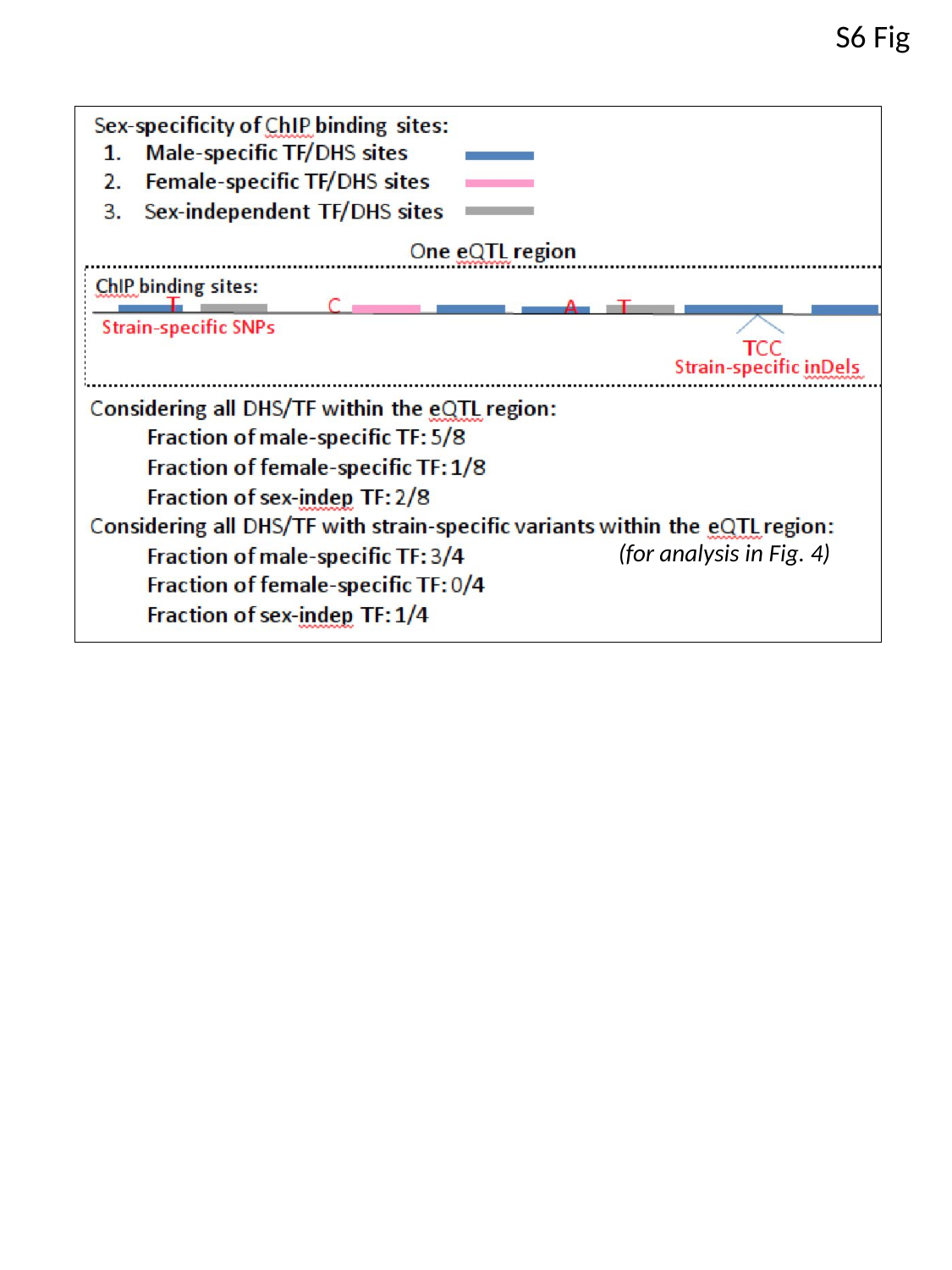

S6 Fig
(for analysis in Fig. 4)

Supplement: S6 Fig — In the example shown, 5 of the 8 regulatory elements (i.e., ChIP-seq binding sites) within the eQTL region shown are male-specific binding sites, three of which contain either SNPs or Indels specific for the regulating strain. A fourth regulatory element with a strain-specific SNP is a sex-independent binding site. One strain-specific SNP is not in a regulatory element, and so is excluded from the analyses shown in Fig 4, as are the four reguatory elements without any strain-specific SNPs/indels. (PPTX) [file pone.0242665.s006.pptx]

## Slide 1
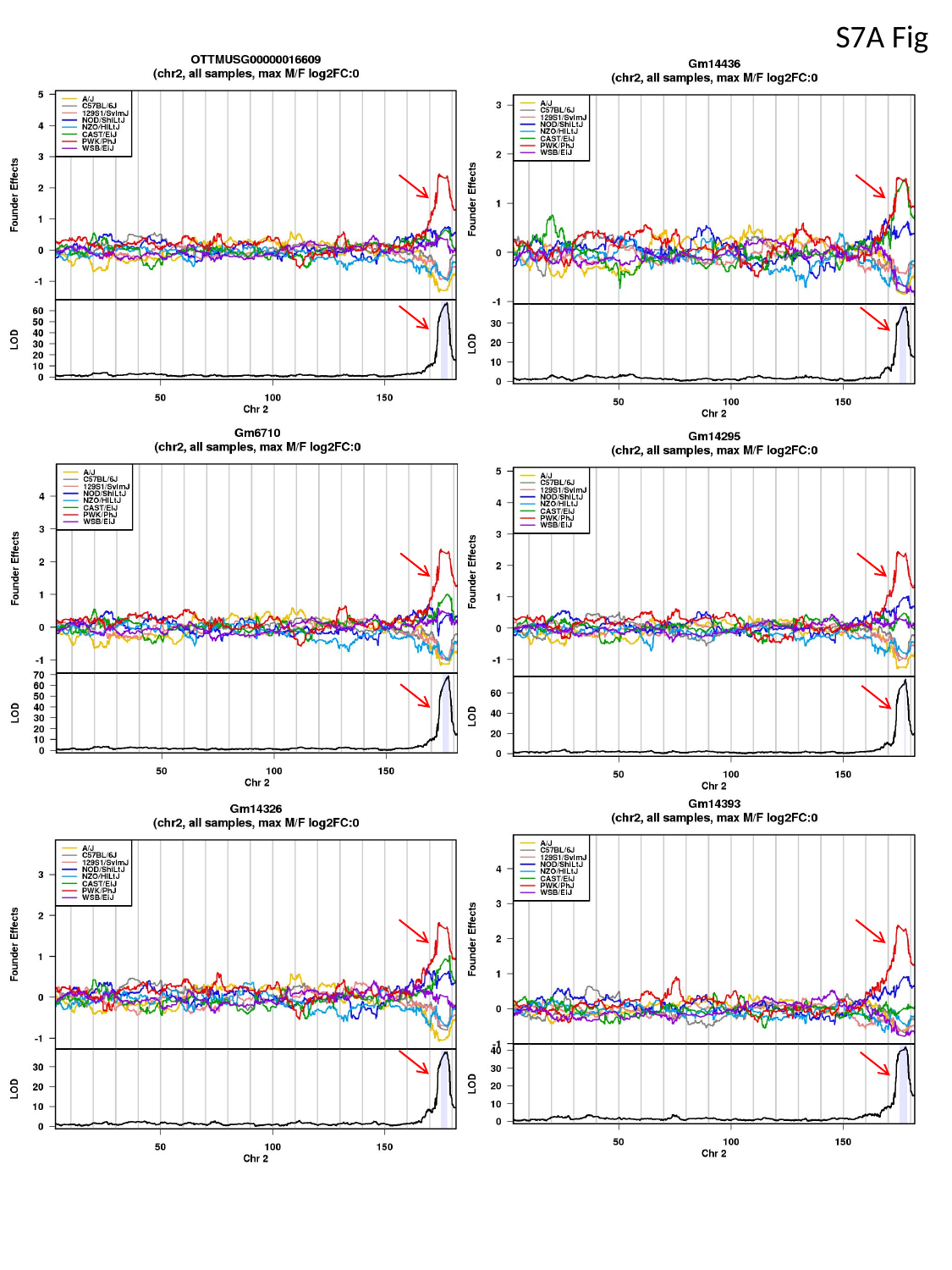

S7A Fig

## Slide 2
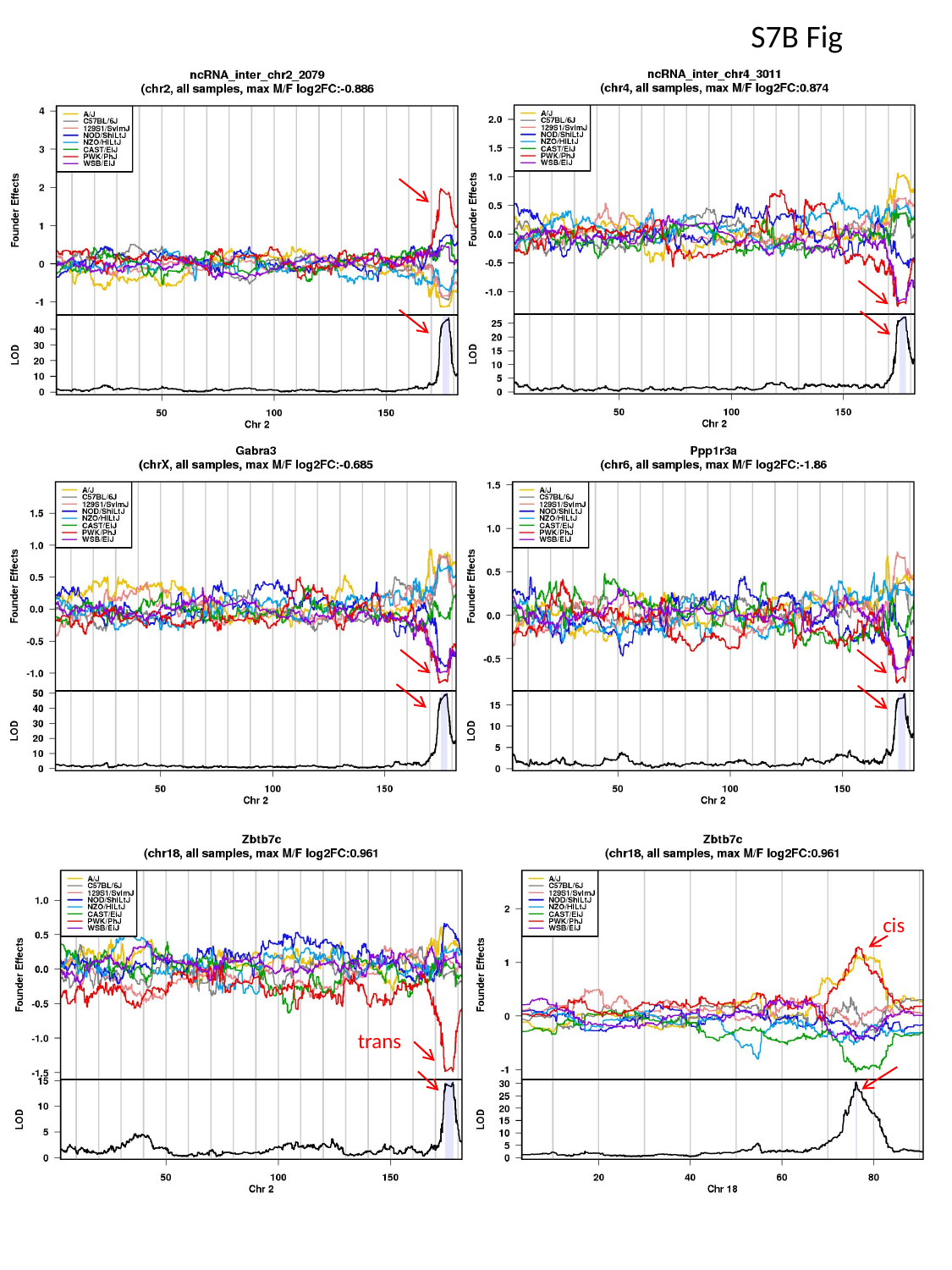

S7B Fig
cis
trans

## Slide 3
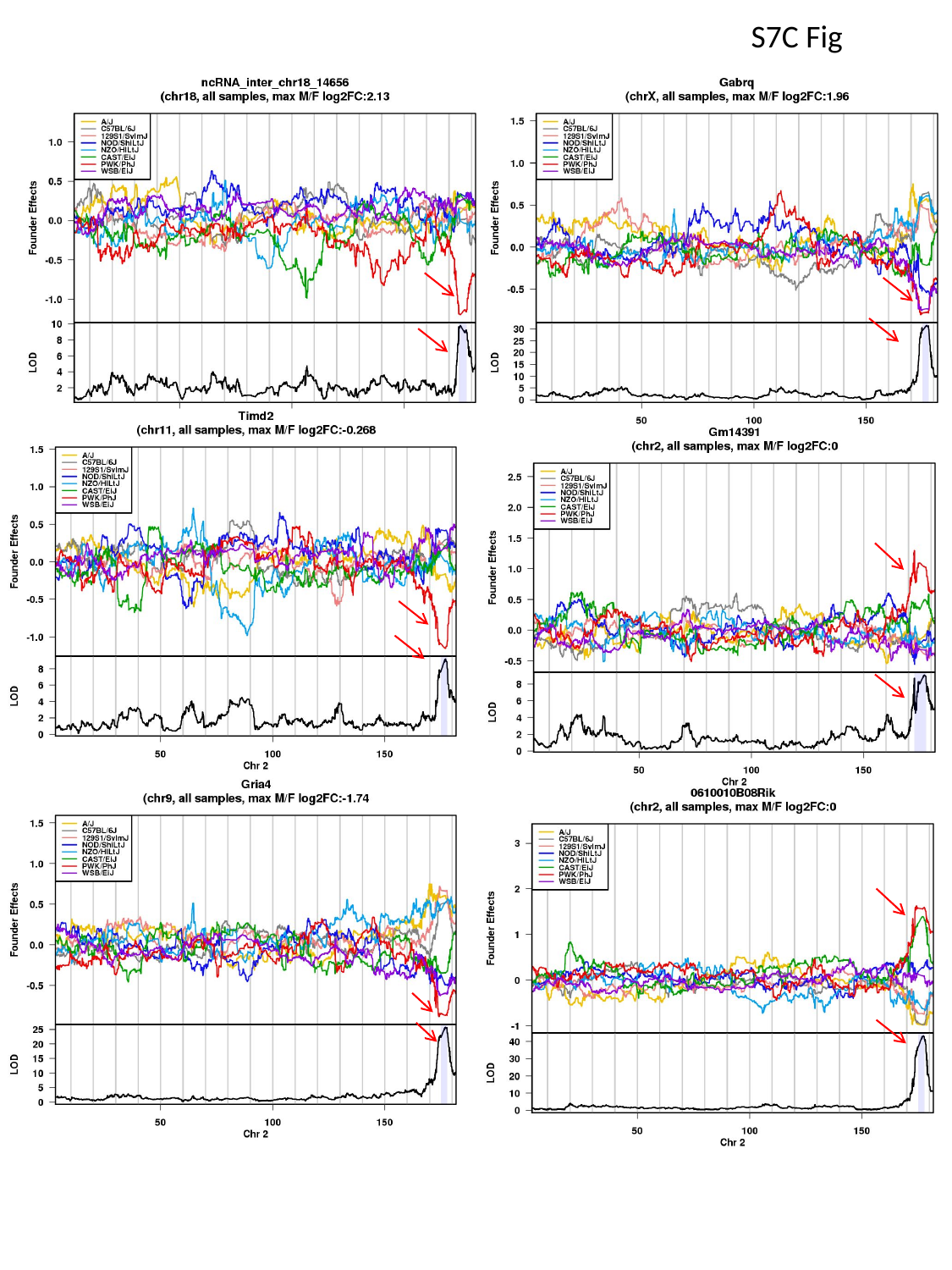

S7C Fig

## Slide 4
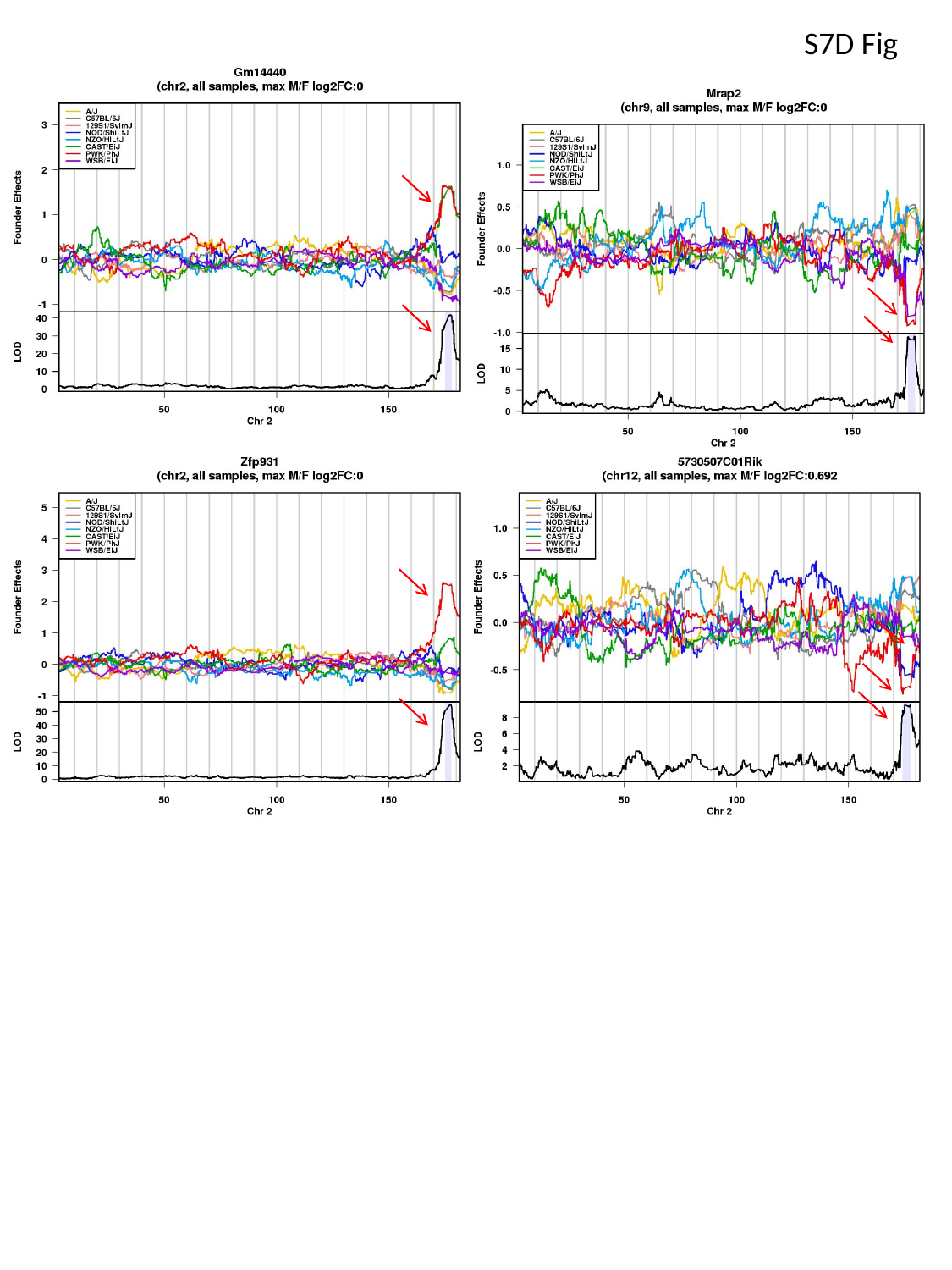

S7D Fig

Supplement: S7 Fig — (PPTX) [file pone.0242665.s007.pptx]

## Slide 1
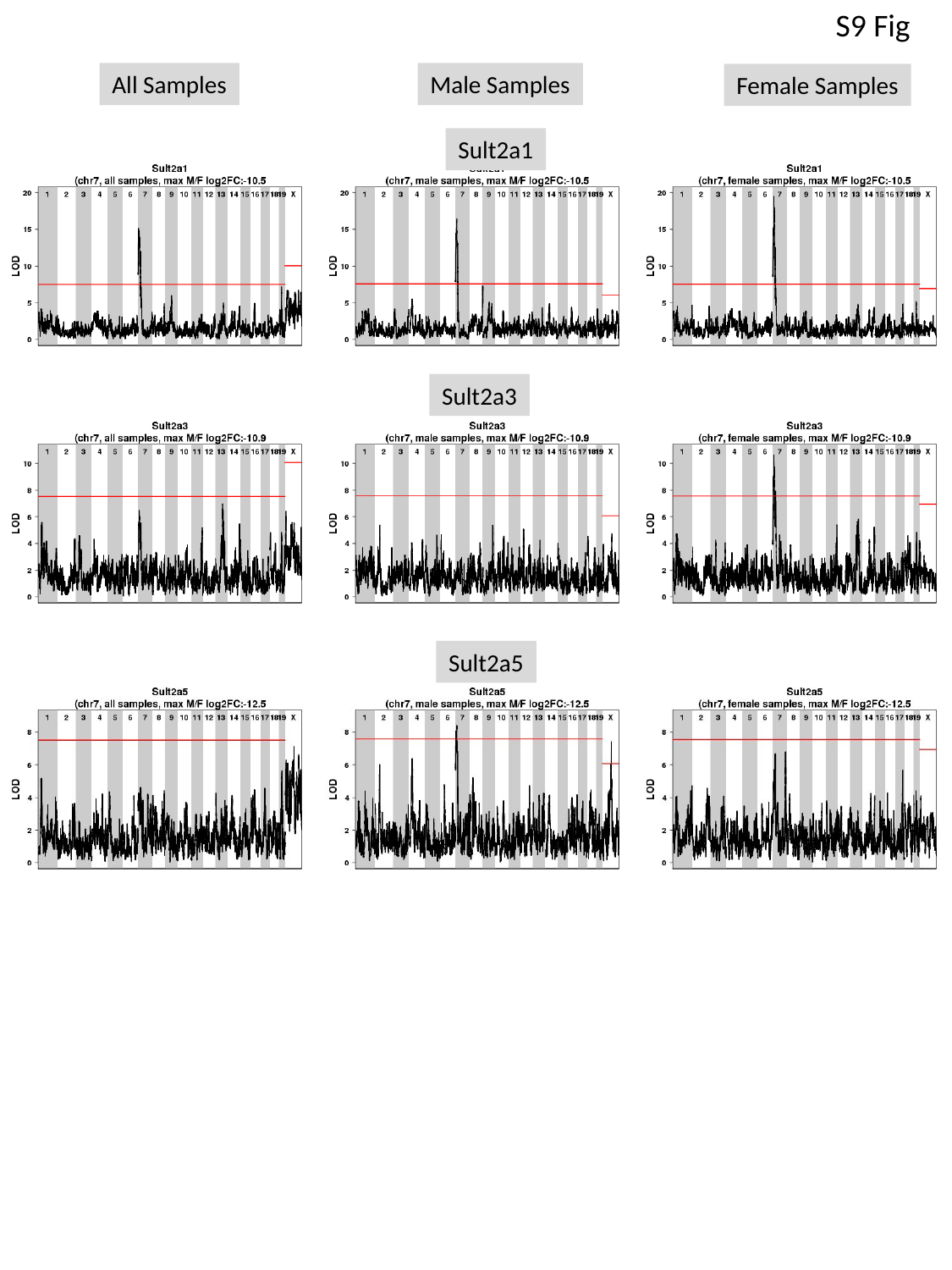

S9 Fig
All Samples
Male Samples
Female Samples
Sult2a1
Sult2a3
Sult2a5

Supplement: S9 Fig — (PPTX) [file pone.0242665.s009.pptx]

## Slide 1
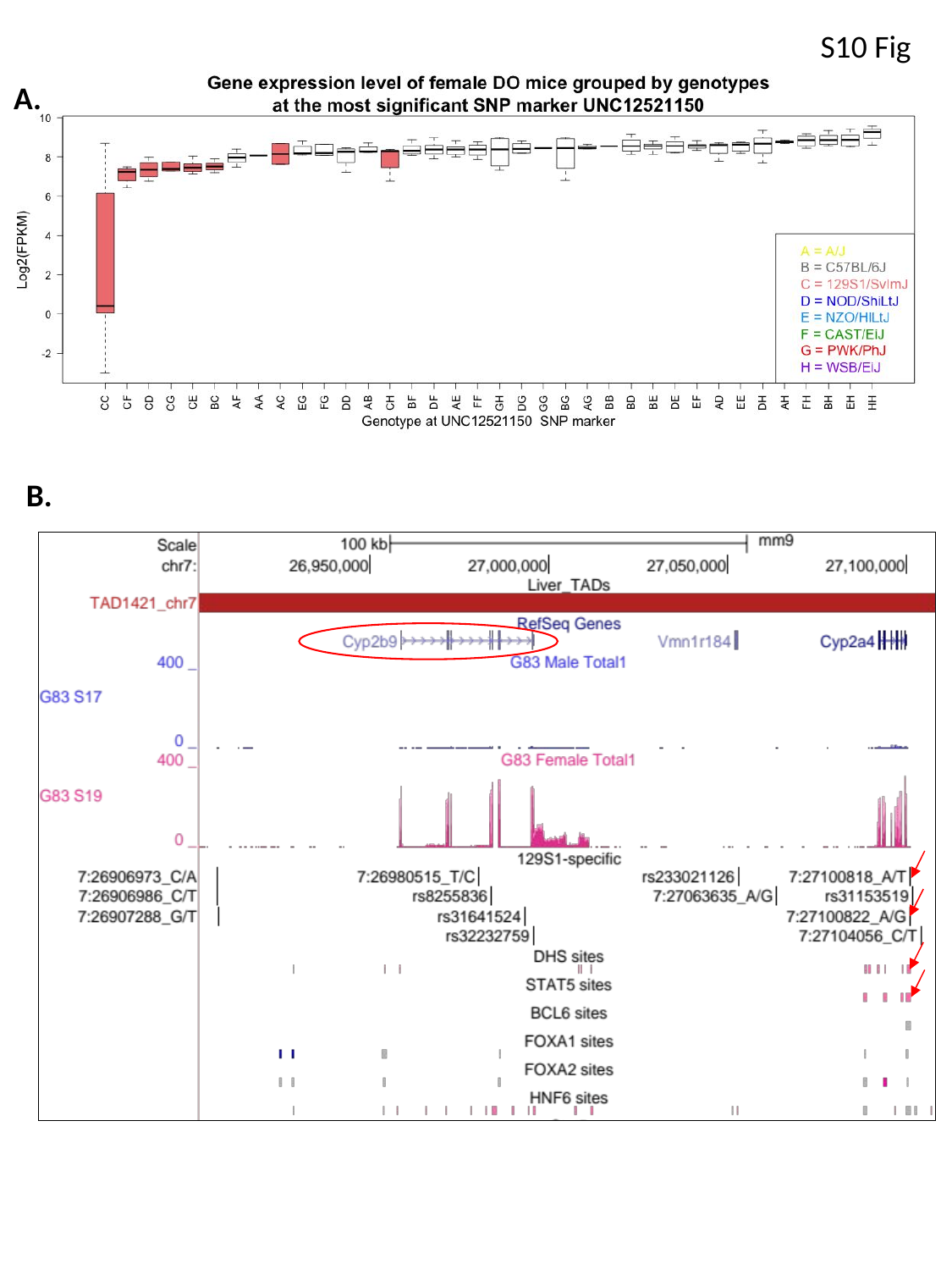

S10 Fig
A.
B.

Supplement: S10 Fig — (A) distribution of gene expression level of Cyp2b9 across individual DO mouse livers (S2 Table) was used to discover eQTLs stratified by the genotype assigned at the the SNP marker with the highest LOD score. (B) A female-biased STAT5 binding sites (fourth red arrow from the top) at a female-biased DHS (third red arrow from the top), containing two 129S1/SvlmJ-specific SNPs/indels (two top arrows), located within the eQTL region for Cyp2b9. Although the 129S1/SvlmJ-specific SNPs/indels in the DHS/STAT5 region (top two red arrows) are closer to Cyp2a4 than Cyp2b9, they are in the same TAD region as Cyp2b9, and more importantly, the eQTL affects the expression of Cyp2b9, but not Cyp2a4. These two genes show a weak negative correlation of their expression in female liver (Pearson correlation = -0.1) and only a moderate correlation in male liver (Pearson correlation = 0.3). (PPTX) [file pone.0242665.s010.pptx]
