# Supplementary material for: Genetic factors contributing to extensive variability of sex-specific hepatic gene expression in Diversity Outbred mice
Source: PLoS One. 2020 Dec 2;15(12):e0242665. doi: 10.1371/journal.pone.0242665 (PMC7710091; doi:10.1371/journal.pone.0242665)
Supplement: S3 Fig — Schematic overview (A), and properties of autosomal eQTLs in DO mouse liver (B). Shown in (B) are the percentages of autosomal eQTLs that are associated with increased expression (white bars) or decreased expression (colored bars) in the respective regulating founder mouse strain. These data are based on 625, 672, 574, 593, 616, 3,085, 3,074 and 1,086 autosomal eQTLs whose regulating strain is A/J, C57BL/6J, 129S1/SvlmJ, NOD/ShiLtJ, NZO/HILtJ, CAST/EiJ, PWK/PhJ, or WSB/EiJ, respectively. Data are based on the 10,325 autosomal eQTLs (Table 1) discovered when the set of all DO liver samples were used for eQTL discovery. (PPTX) [file pone.0242665.s003.pptx]

## Slide 1
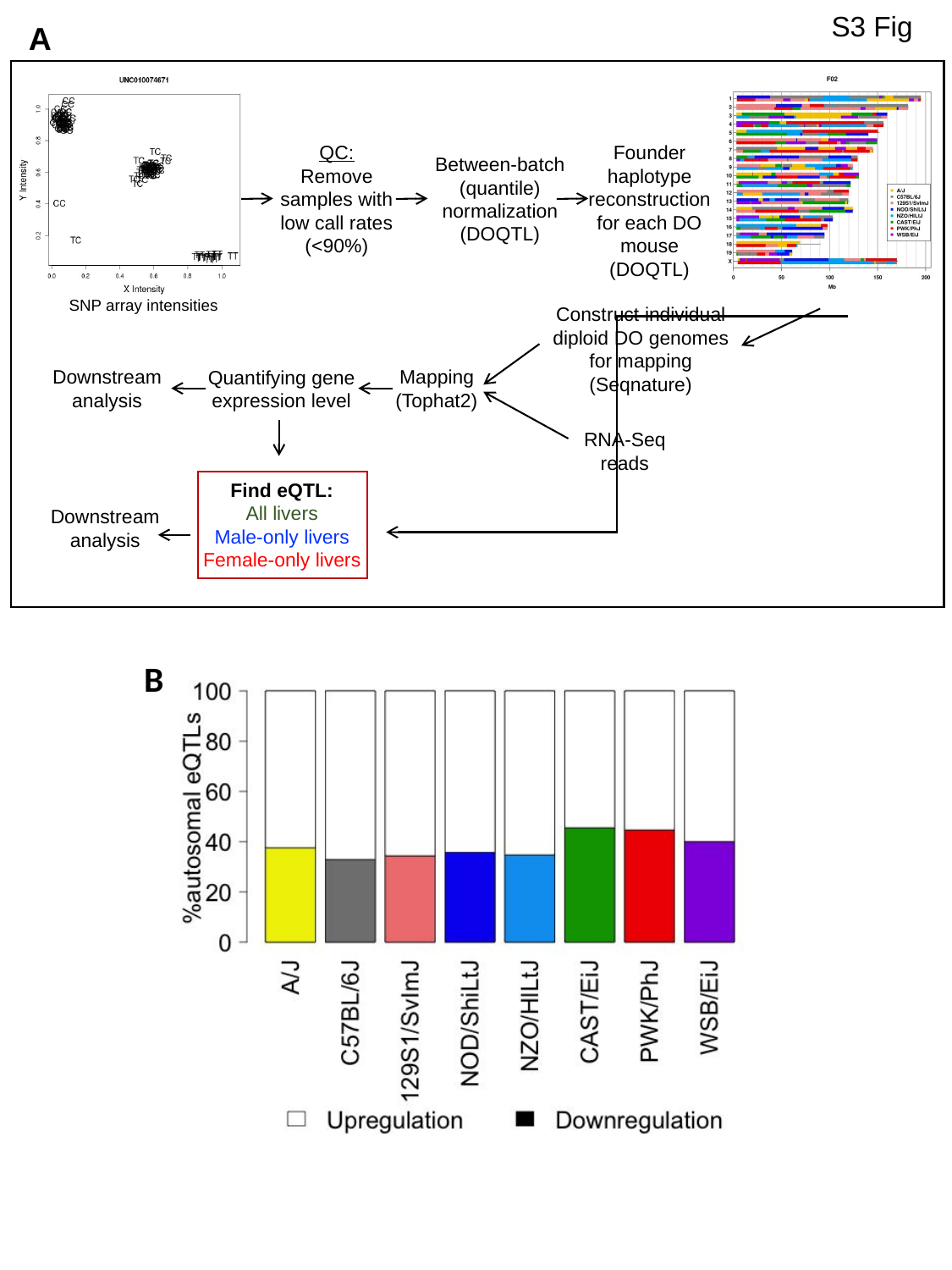

S3 Fig
A
Founder haplotype reconstruction for each DO mouse (DOQTL)
QC:
Remove samples with low call rates (<90%)
Between-batch (quantile) normalization (DOQTL)
SNP array intensities
Construct individual diploid DO genomes for mapping (Seqnature)
Downstream analysis
Mapping (Tophat2)
Quantifying gene expression level
RNA-Seq reads
Find eQTL:
All livers
Male-only livers
Female-only livers
Downstream analysis
B
1
