# Supplementary material for: Genetic factors contributing to extensive variability of sex-specific hepatic gene expression in Diversity Outbred mice
Source: PLoS One. 2020 Dec 2;15(12):e0242665. doi: 10.1371/journal.pone.0242665 (PMC7710091; doi:10.1371/journal.pone.0242665)
Supplement: S4 Fig — For each gene, the figure shows the gene expression patterns across strains (see S1 Fig for details), and genome-wide eQTL scans using the three different DO liver datasets, where the horizontal red line marks the P < 0.05 significance cutoff for eQTL significance based on the permutation test. Also shown are regression coefficients across the chromosome that contains the significant eQTL peak, as follows: Regression coefficients (top of each panel) and LOD scores (log10(p-value); bottom of each panel) across the chromosome that has a significant eQTL peak in male (left) or female (right) mouse liver, as marked at bottom. Shaded area in the LOD score plot indicates the 95% Bayesian credible interval for each eQTL. (A) Cav1 shows strong male-specific expression in the PWK/PhJ founder strain and in a subset of male DO mouse livers. Its strong eQTL is seen in male livers only and is associated with the PWK/PhJ strain. (B) Hsd3b5 shows male-biased expression in CD-1 mice and multiple DO founder strains; its expression is repressed in female C57BL/6J mice. (PPTX) [file pone.0242665.s004.pptx]

## Slide 1
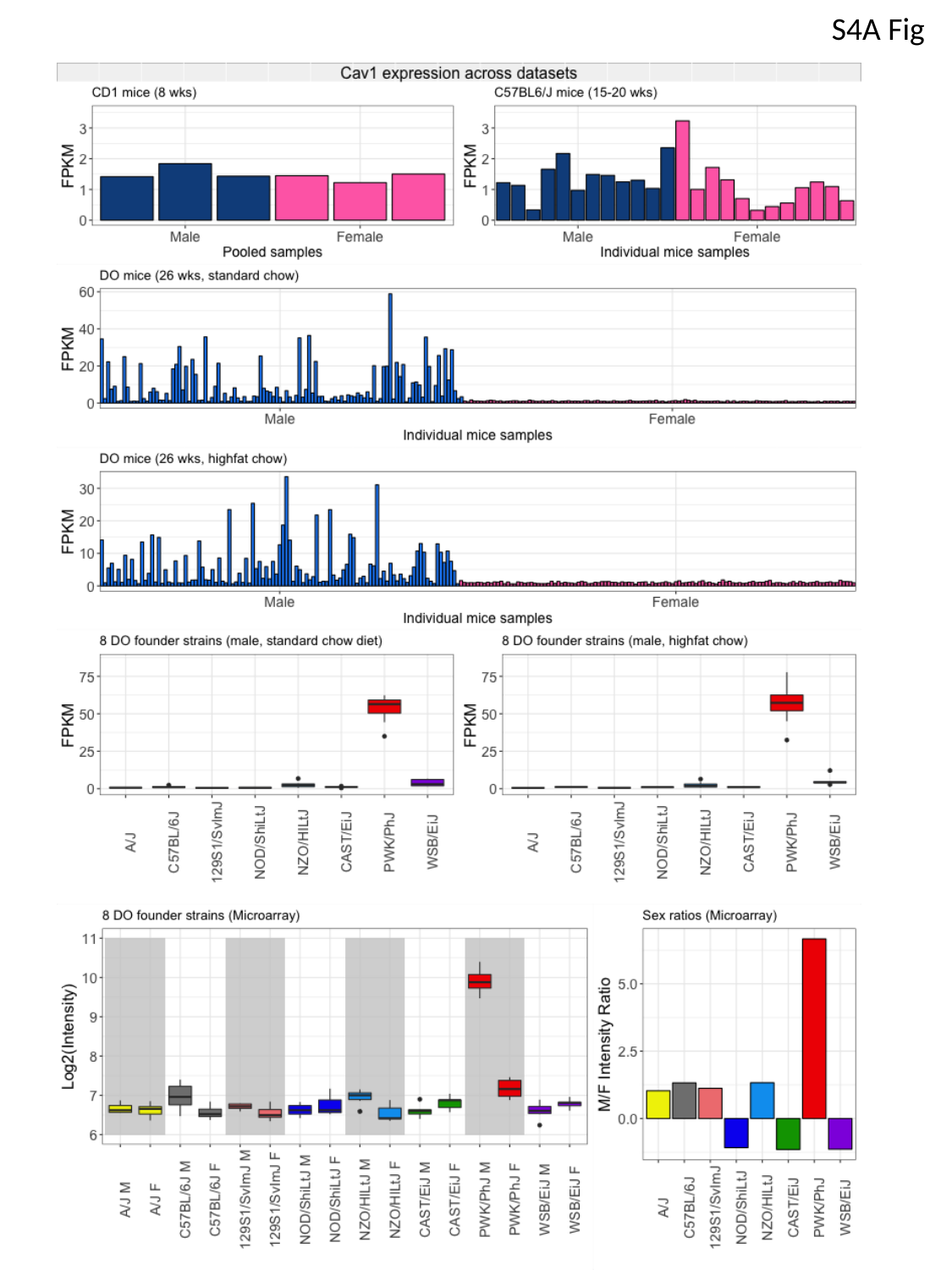

S4A Fig

## Slide 2
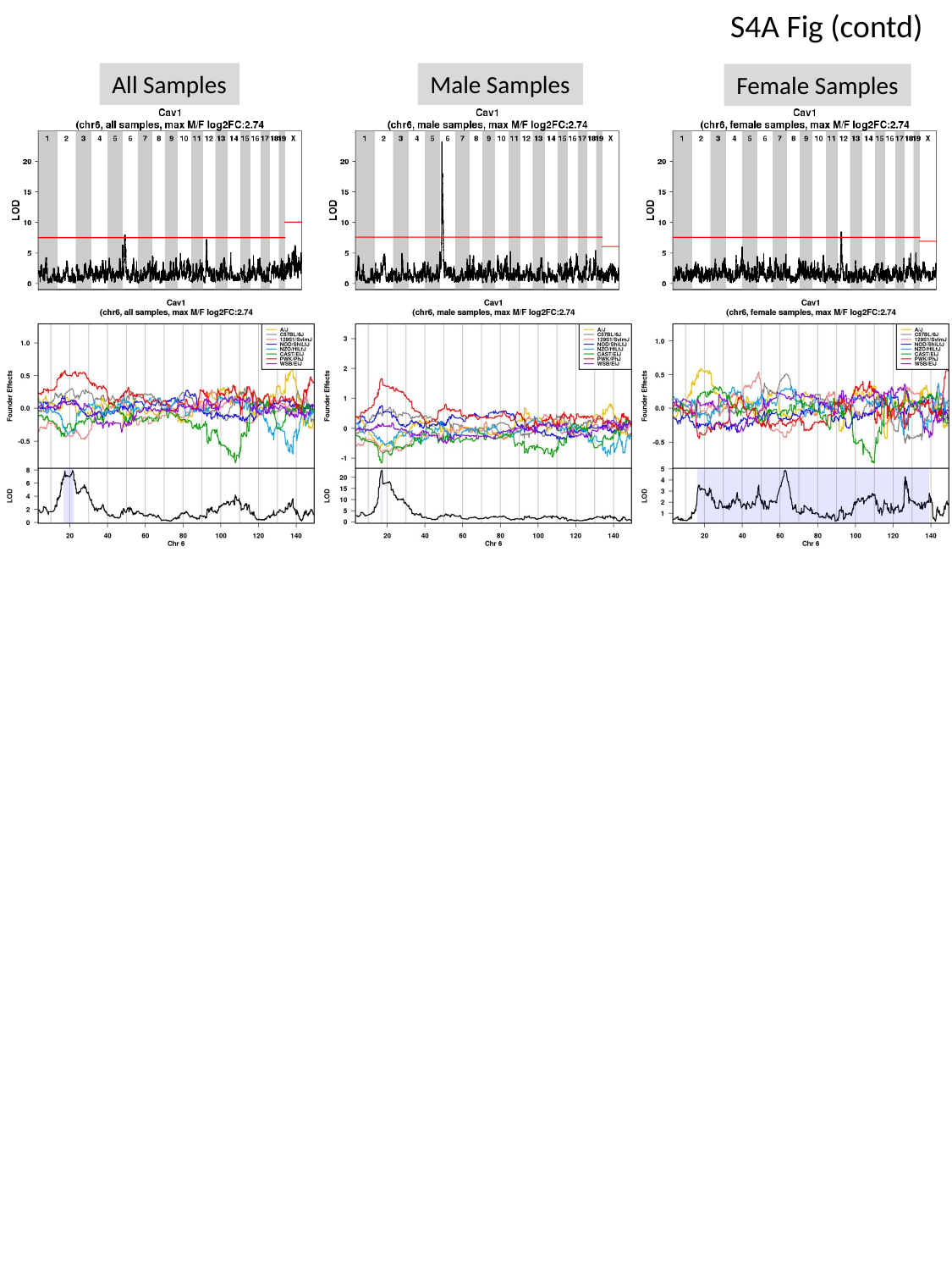

S4A Fig (contd)
All Samples
Male Samples
Female Samples

## Slide 3
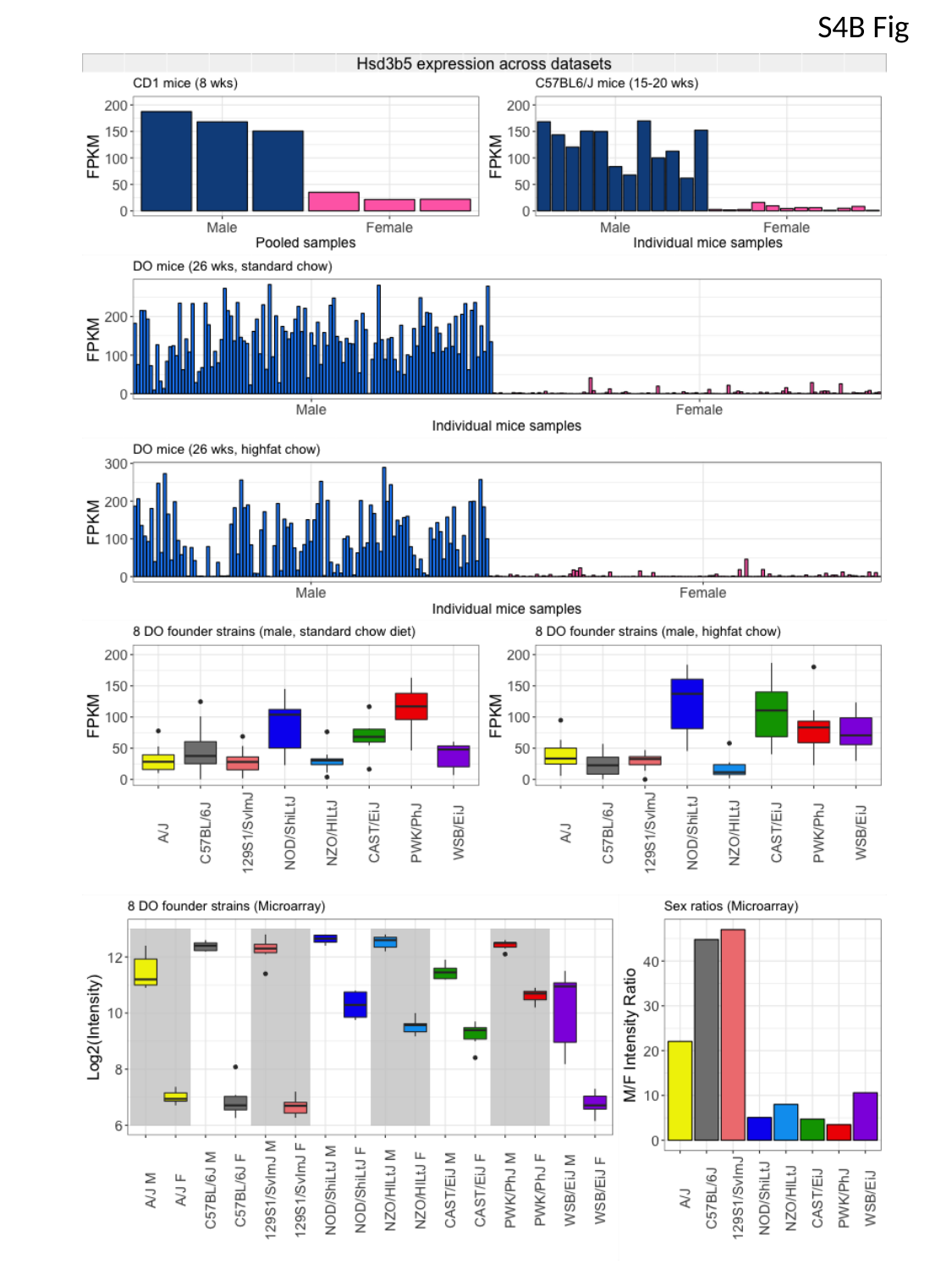

S4B Fig

## Slide 4
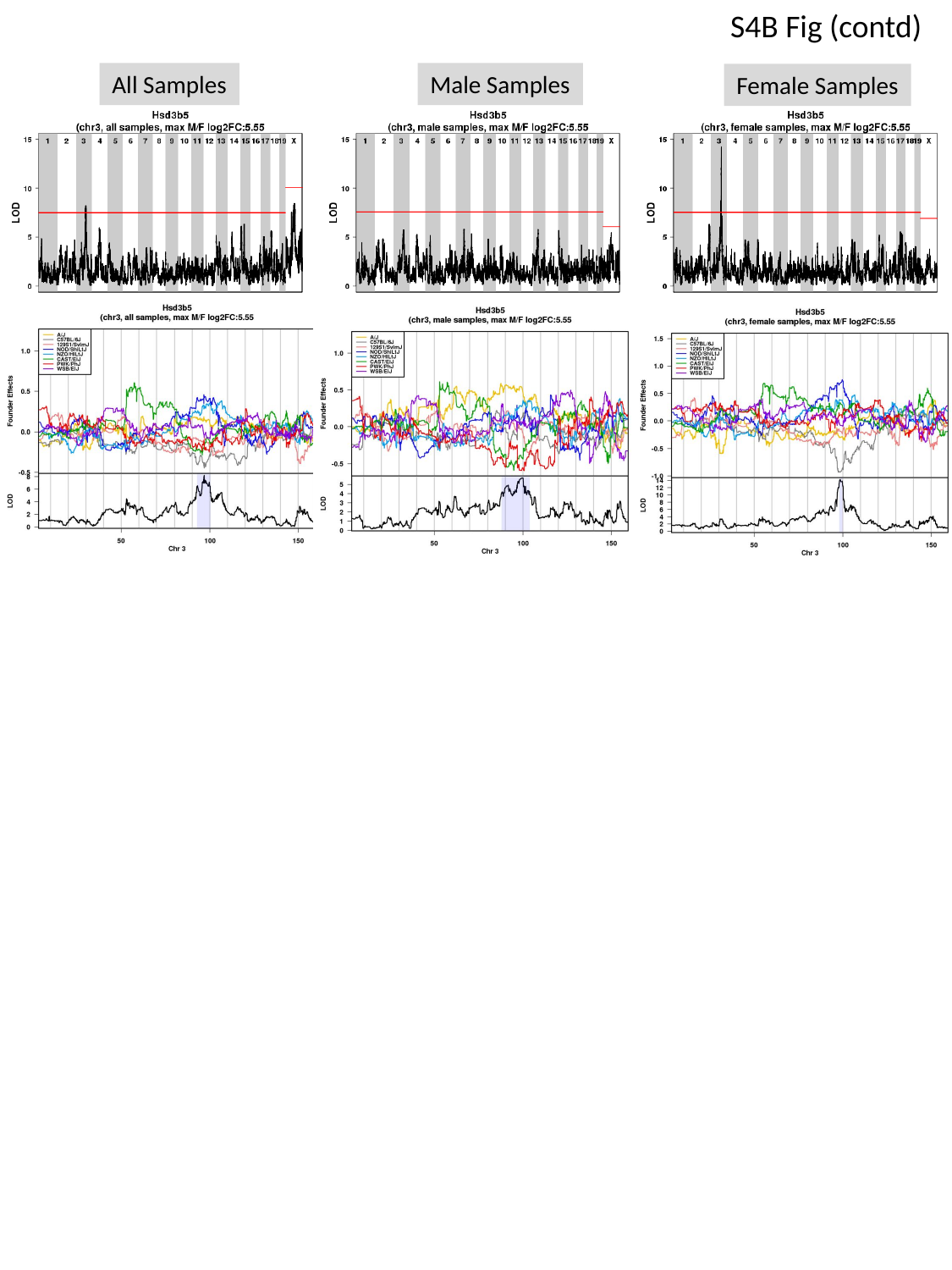

S4B Fig (contd)
All Samples
Male Samples
Female Samples
