# Supplementary material for: Genetic factors contributing to extensive variability of sex-specific hepatic gene expression in Diversity Outbred mice
Source: PLoS One. 2020 Dec 2;15(12):e0242665. doi: 10.1371/journal.pone.0242665 (PMC7710091; doi:10.1371/journal.pone.0242665)
Supplement: S5 Fig — For each gene, the figure shows gene expression patterns across strains, genome-wide eQTL scan, and regression coefficients at the chromosome with a significant peak, as described in the legend to S1 Fig. (PPTX) [file pone.0242665.s005.pptx]

## Slide 1
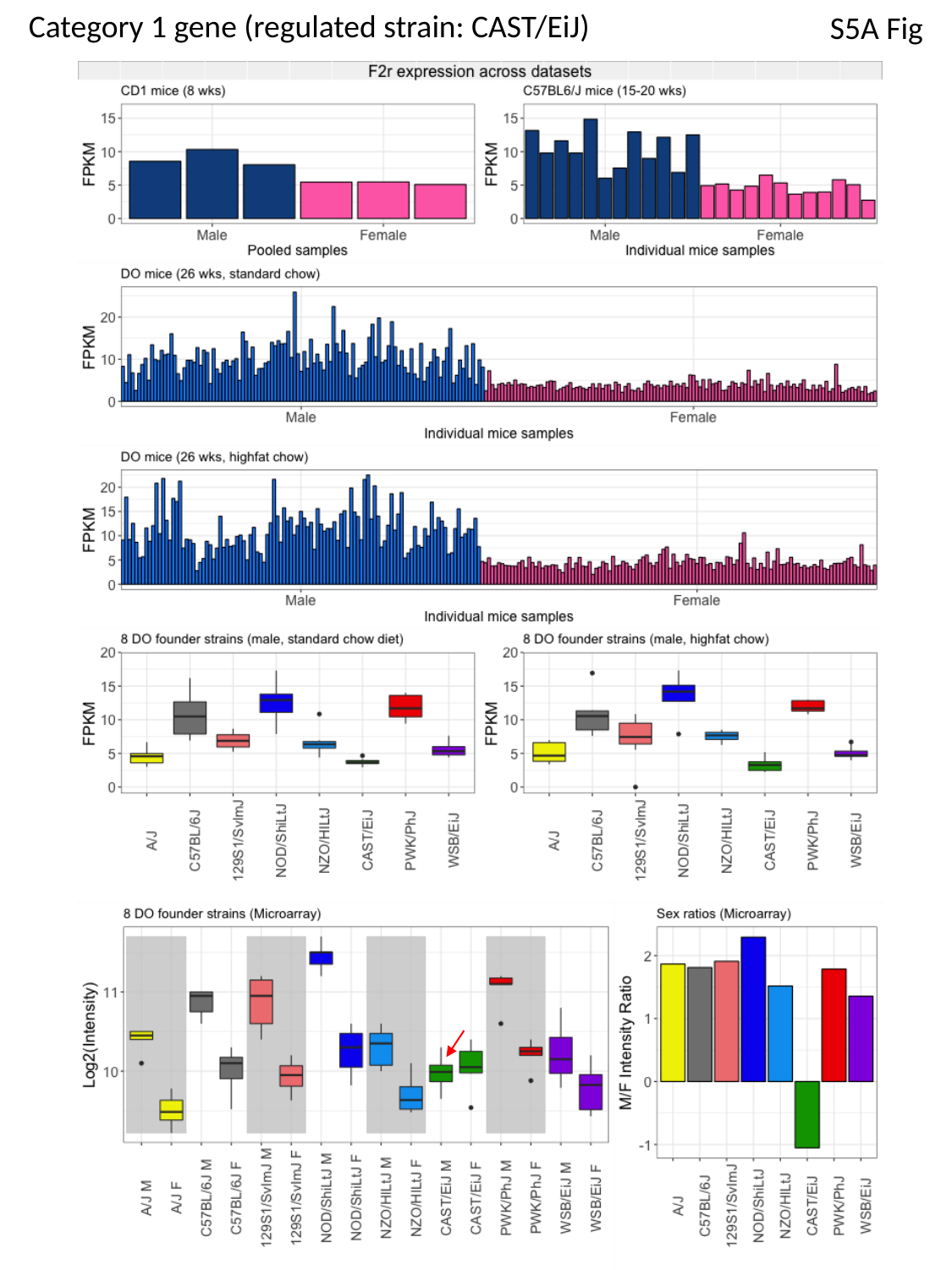

Category 1 gene (regulated strain: CAST/EiJ)
S5A Fig

## Slide 2
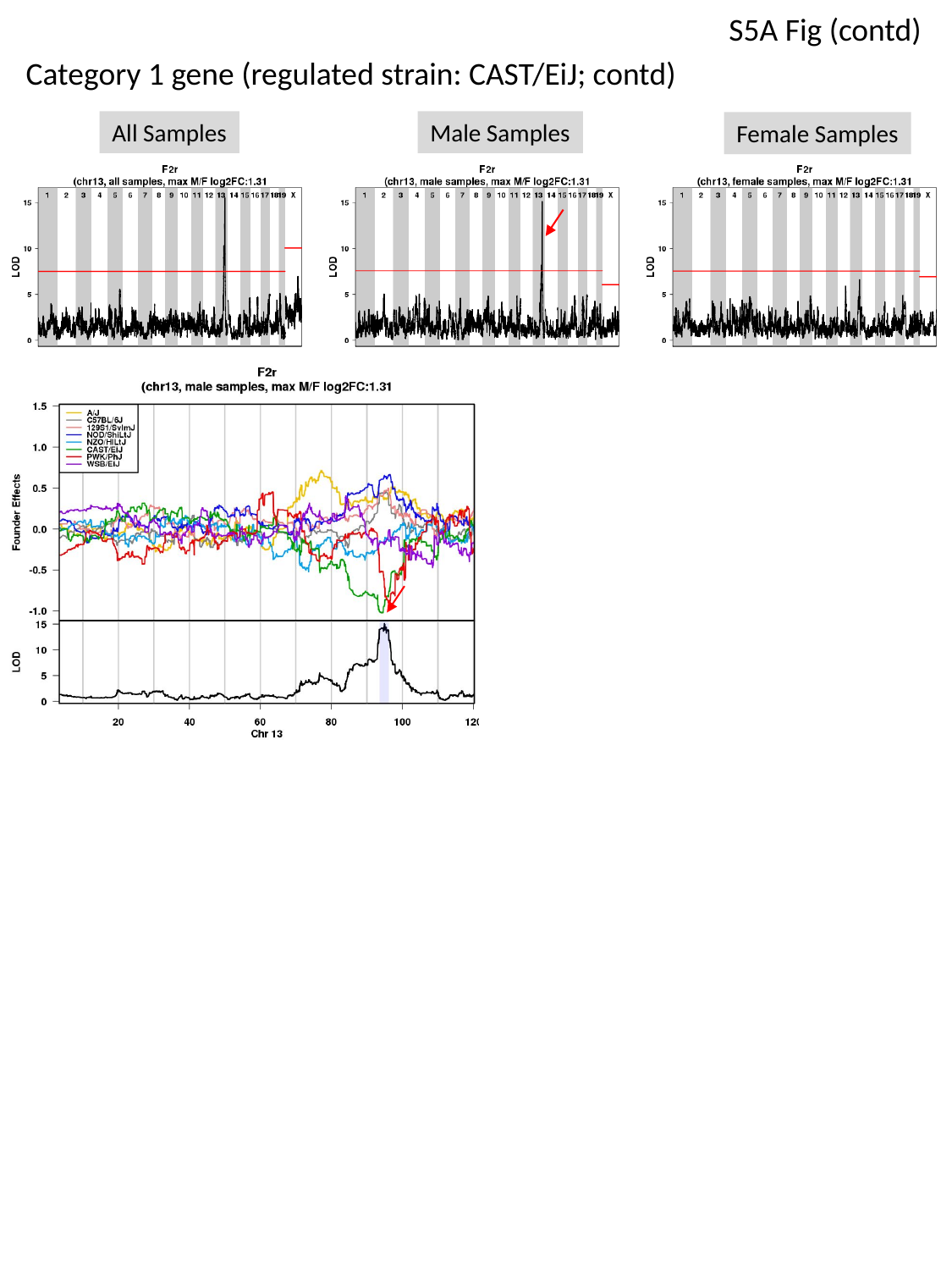

S5A Fig (contd)
Category 1 gene (regulated strain: CAST/EiJ; contd)
All Samples
Male Samples
Female Samples

## Slide 3
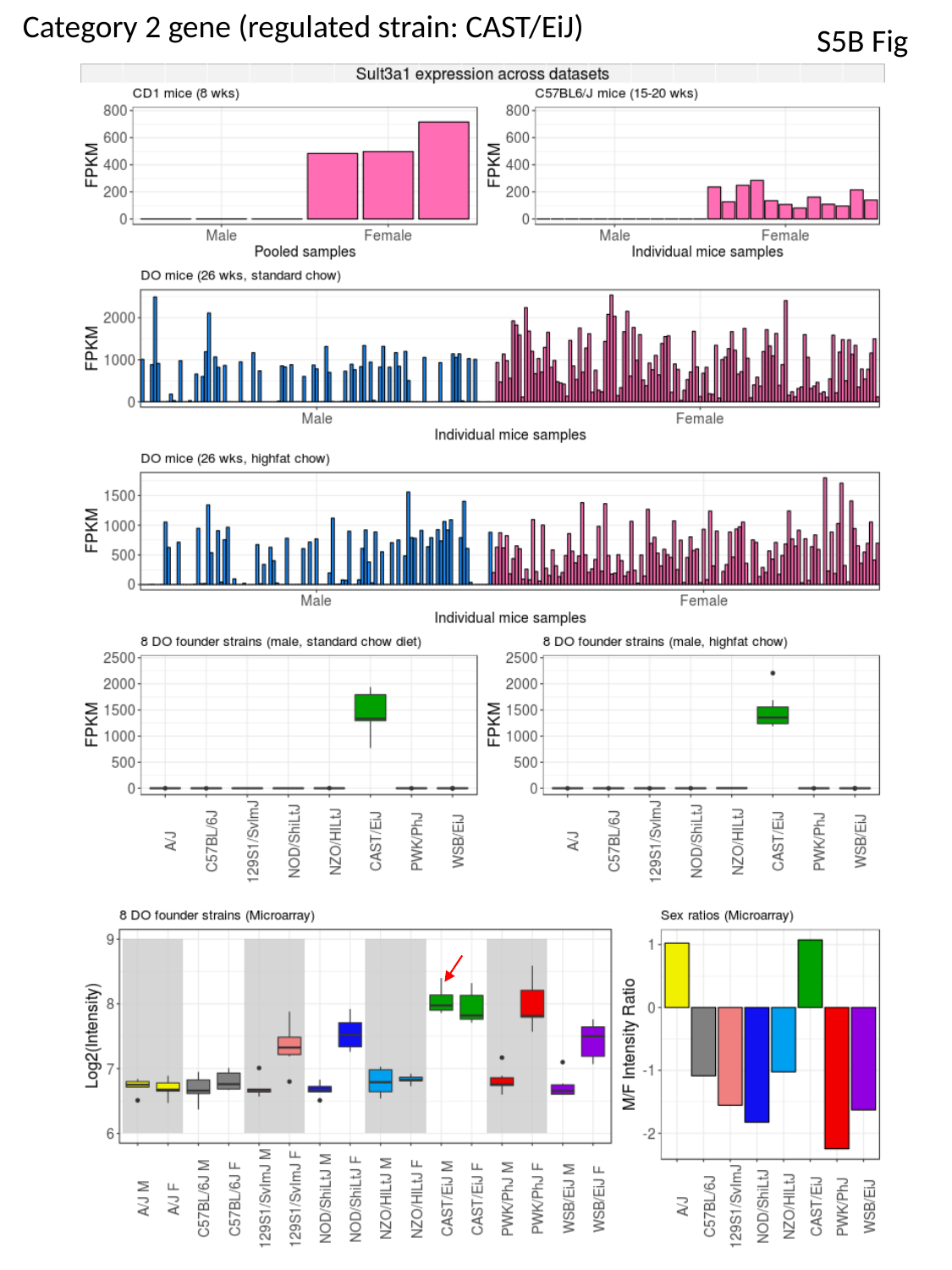

Category 2 gene (regulated strain: CAST/EiJ)
S5B Fig

## Slide 4
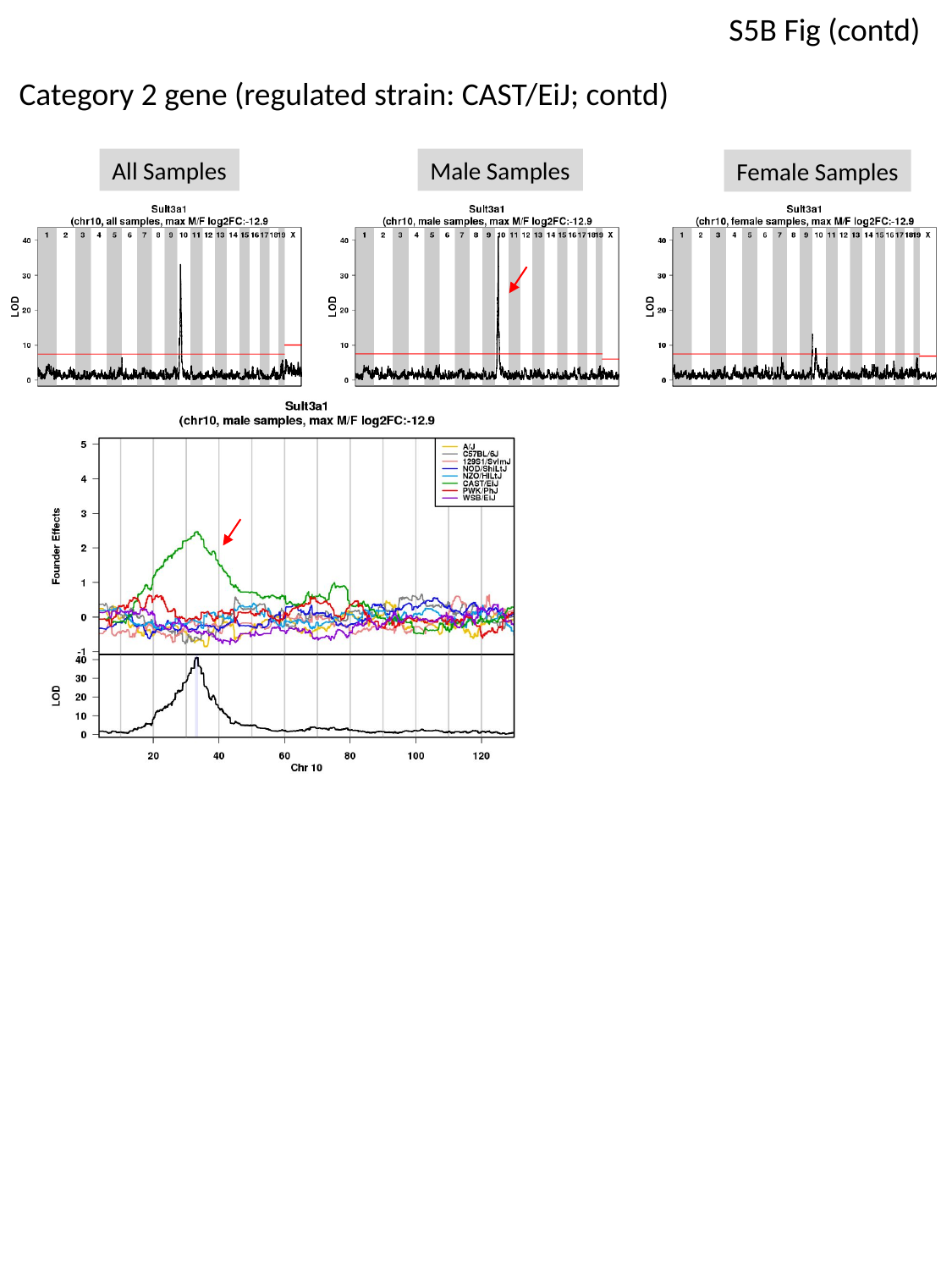

S5B Fig (contd)
Category 2 gene (regulated strain: CAST/EiJ; contd)
All Samples
Male Samples
Female Samples

## Slide 5
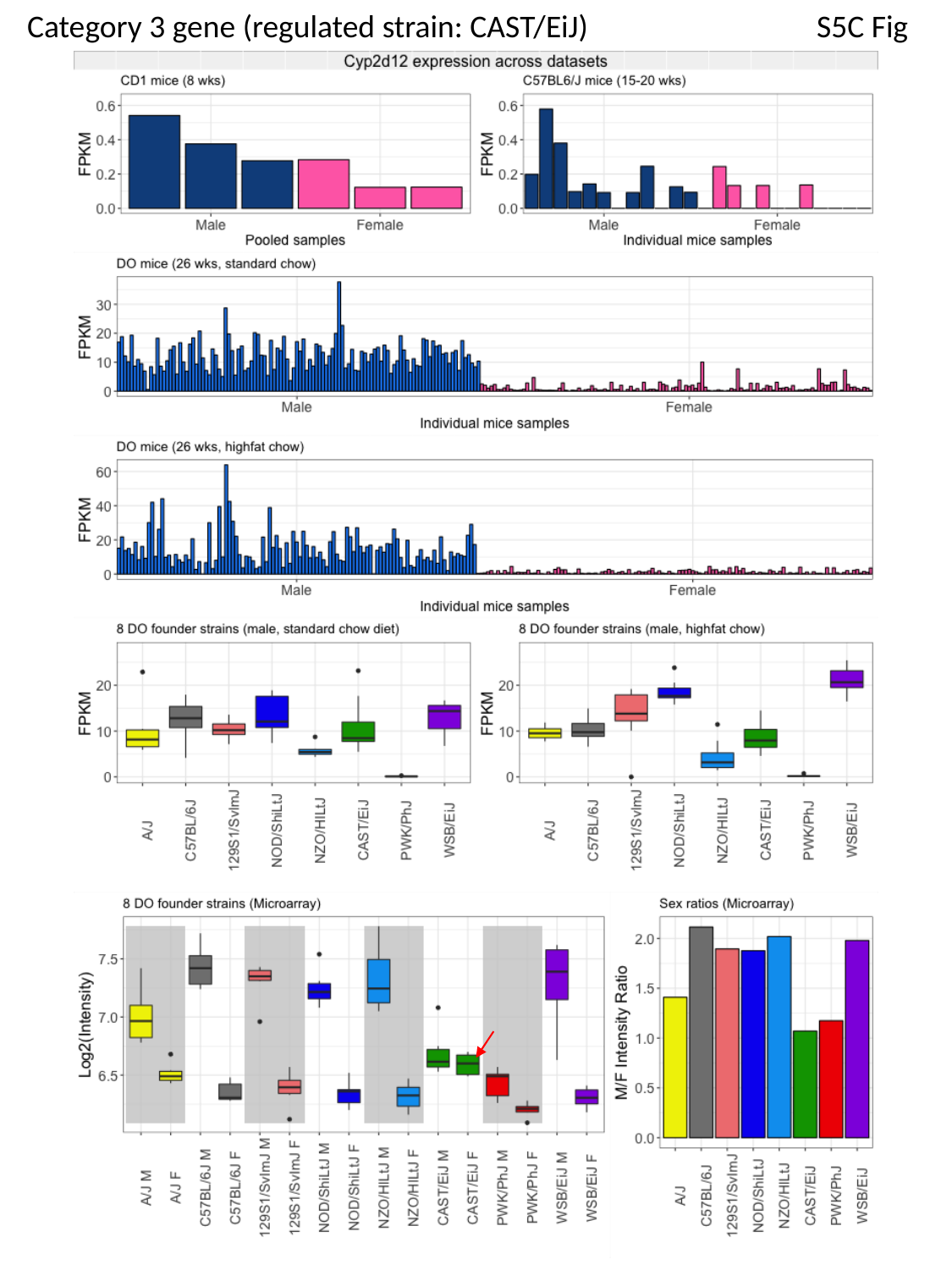

Category 3 gene (regulated strain: CAST/EiJ)
S5C Fig

## Slide 6
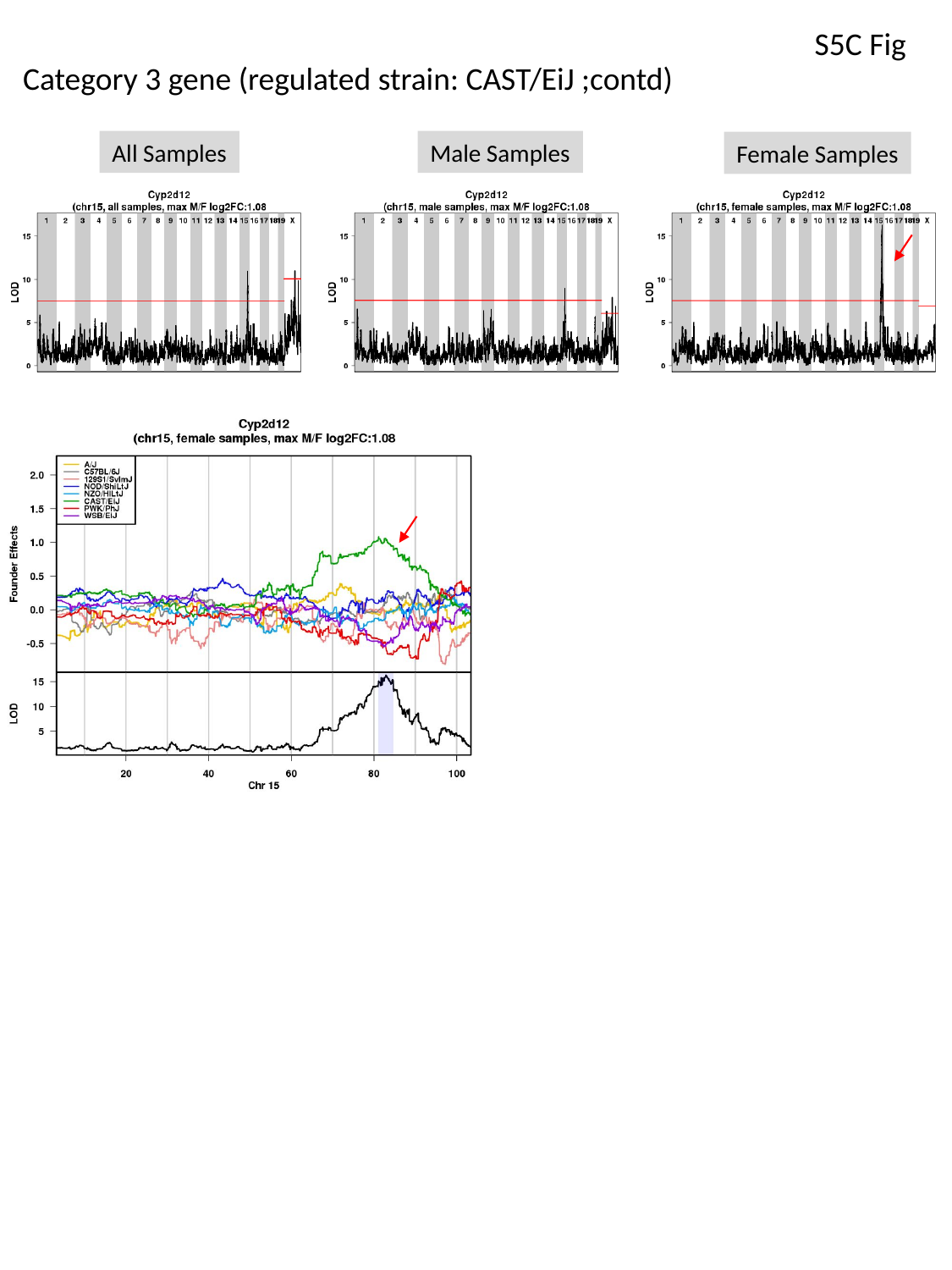

S5C Fig
Category 3 gene (regulated strain: CAST/EiJ ;contd)
All Samples
Male Samples
Female Samples

## Slide 7
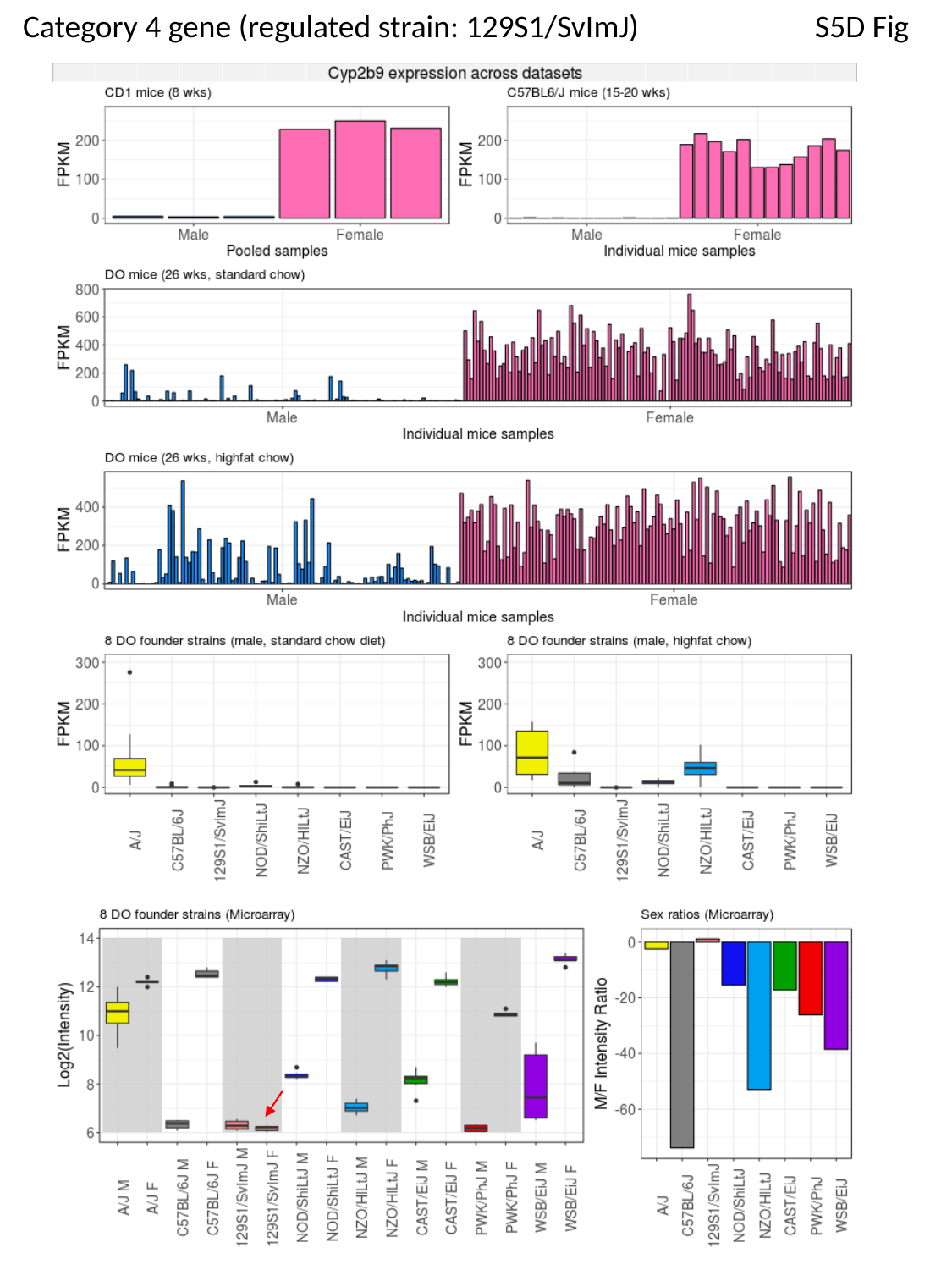

Category 4 gene (regulated strain: 129S1/SvImJ)
S5D Fig

## Slide 8
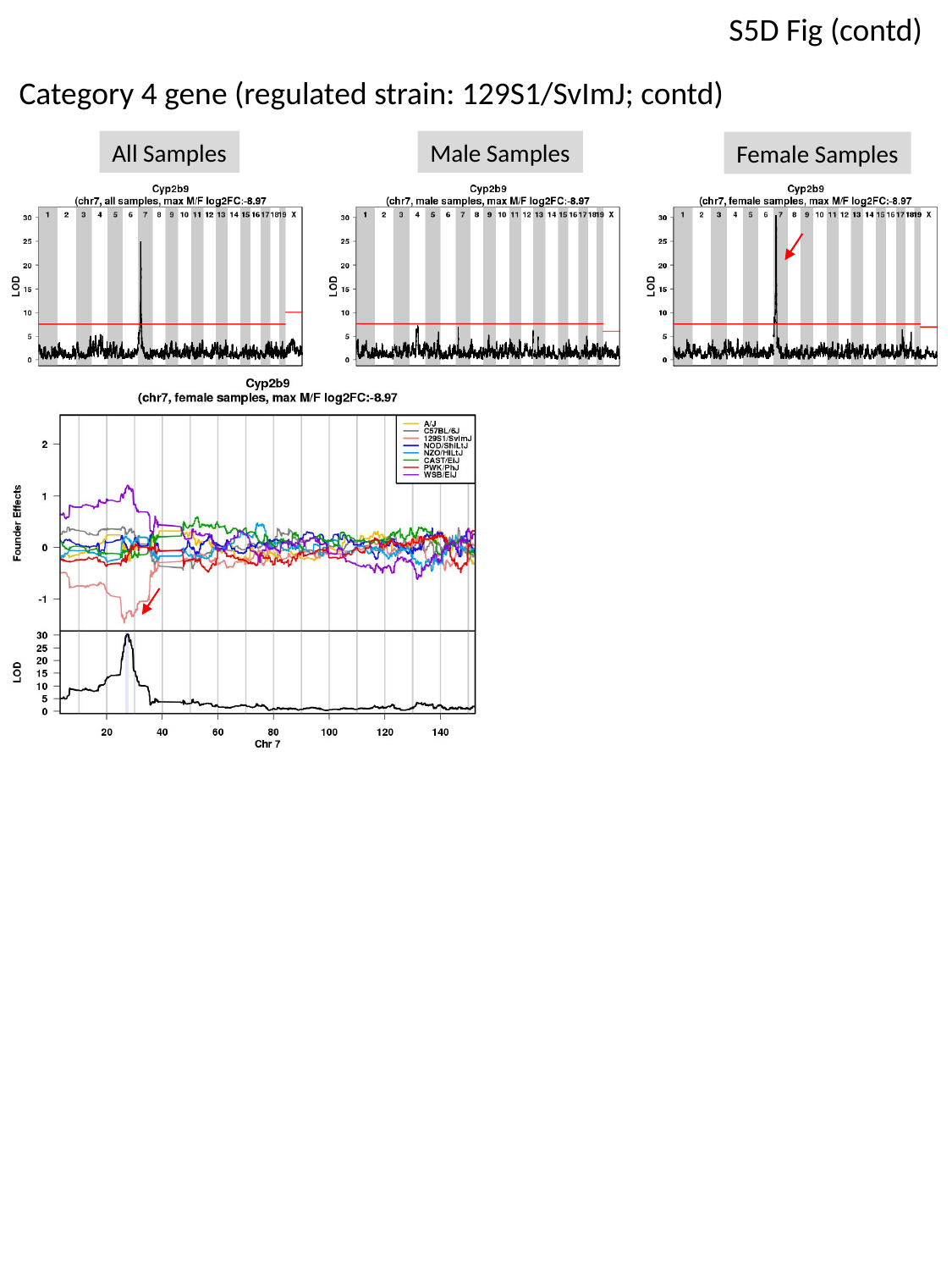

S5D Fig (contd)
Category 4 gene (regulated strain: 129S1/SvImJ; contd)
All Samples
Male Samples
Female Samples

## Slide 9
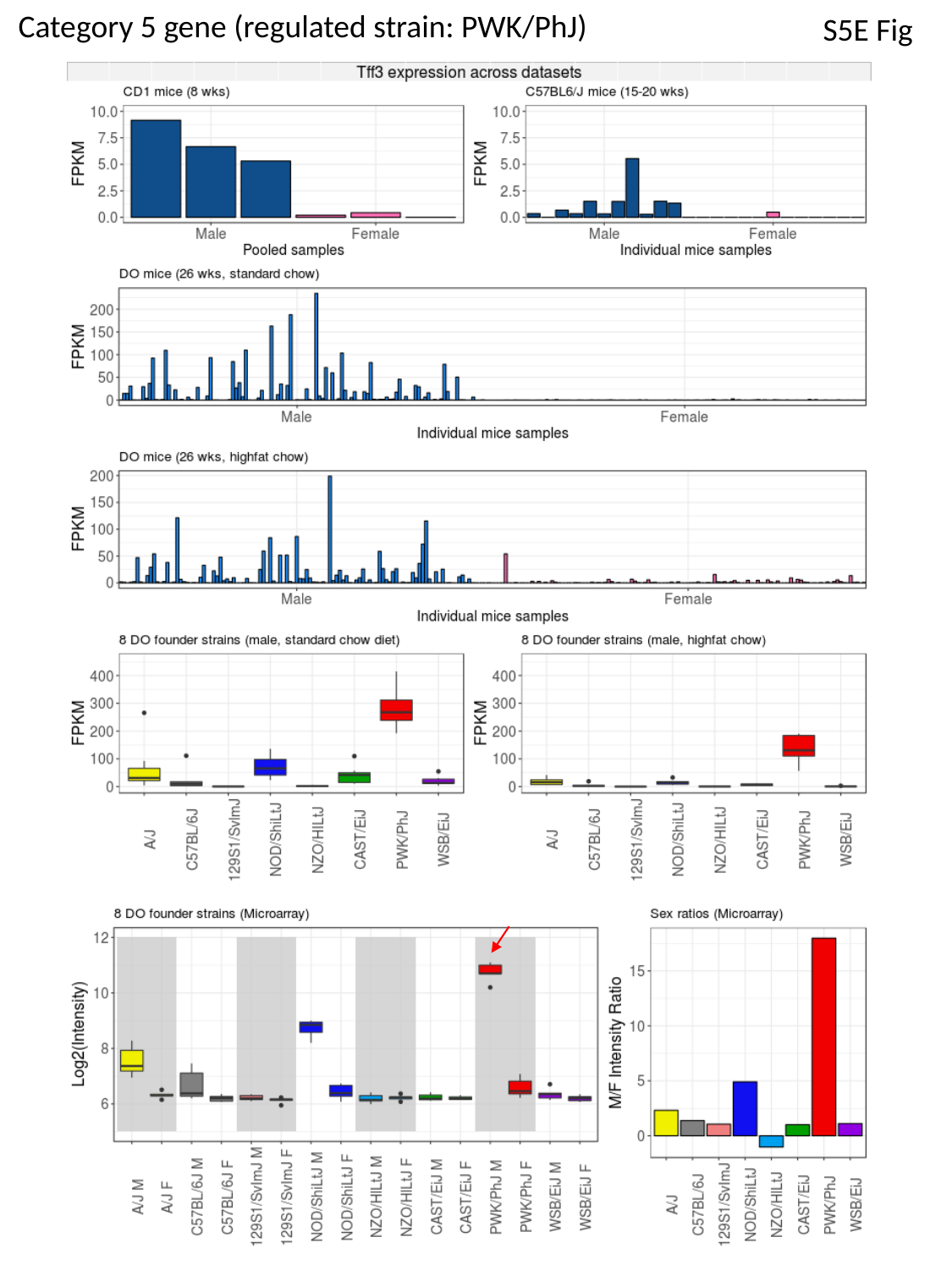

Category 5 gene (regulated strain: PWK/PhJ)
S5E Fig

## Slide 10
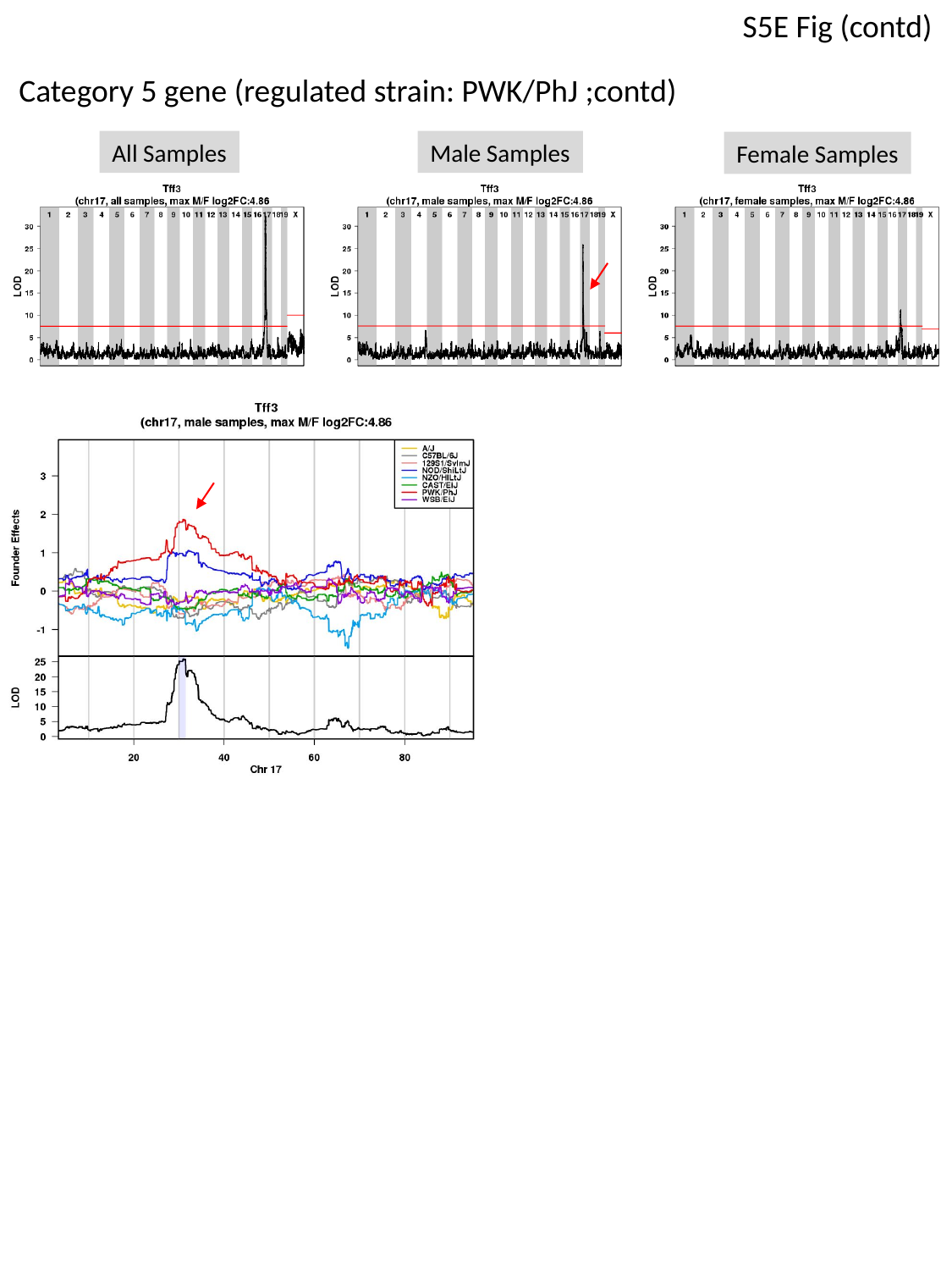

S5E Fig (contd)
Category 5 gene (regulated strain: PWK/PhJ ;contd)
All Samples
Male Samples
Female Samples

## Slide 11
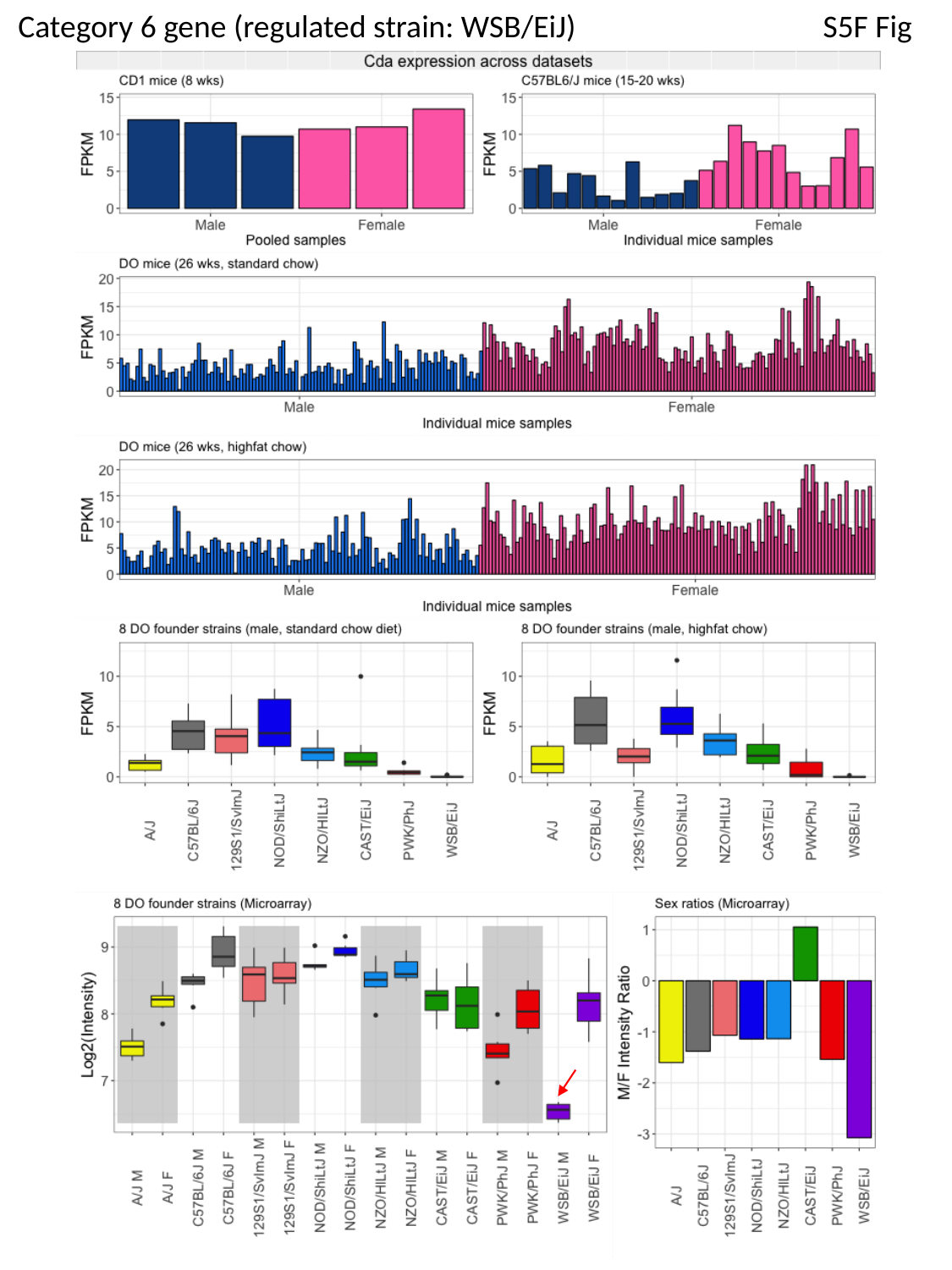

Category 6 gene (regulated strain: WSB/EiJ)
S5F Fig

## Slide 12
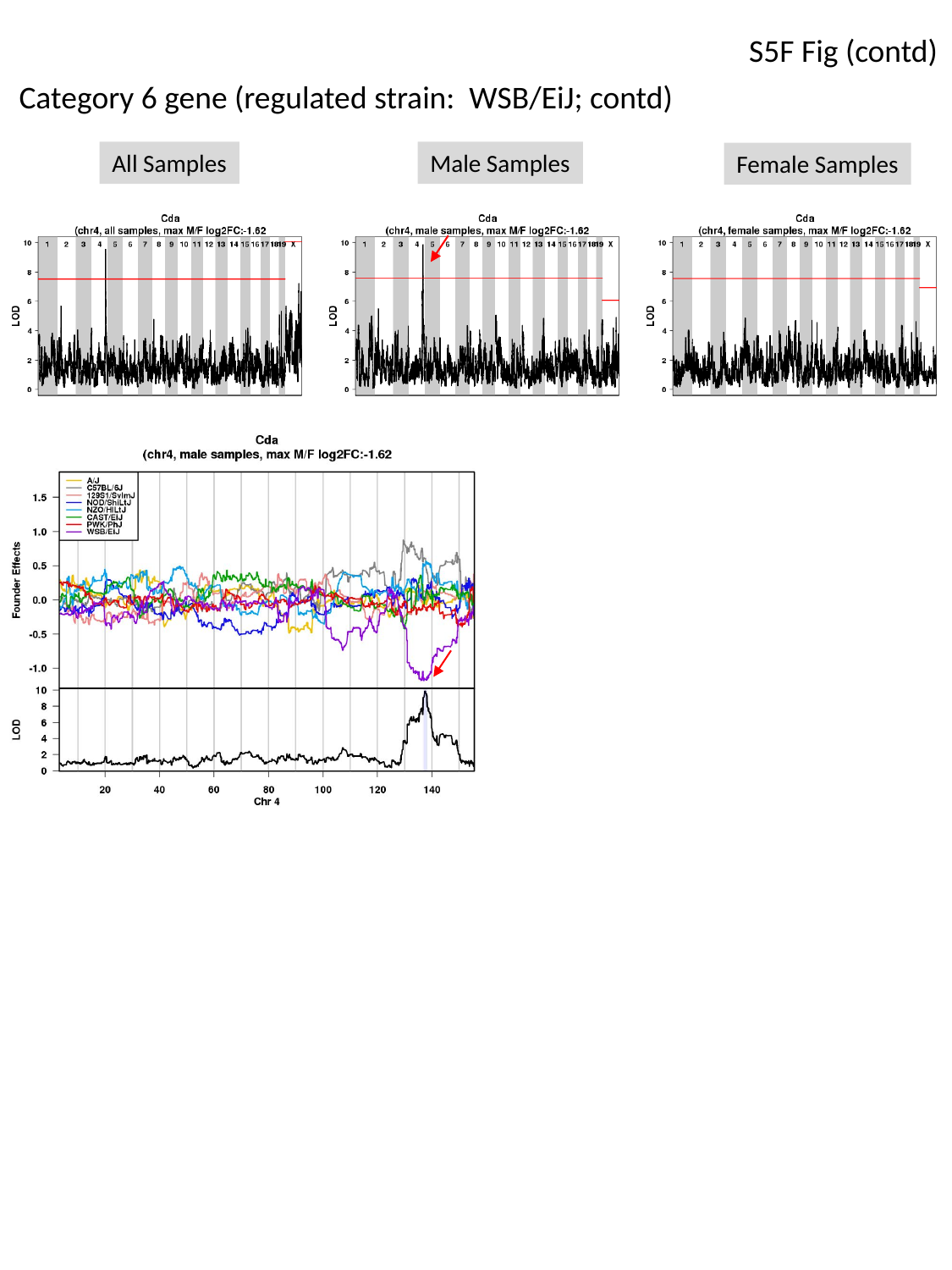

S5F Fig (contd)
Category 6 gene (regulated strain: WSB/EiJ; contd)
All Samples
Male Samples
Female Samples

## Slide 13
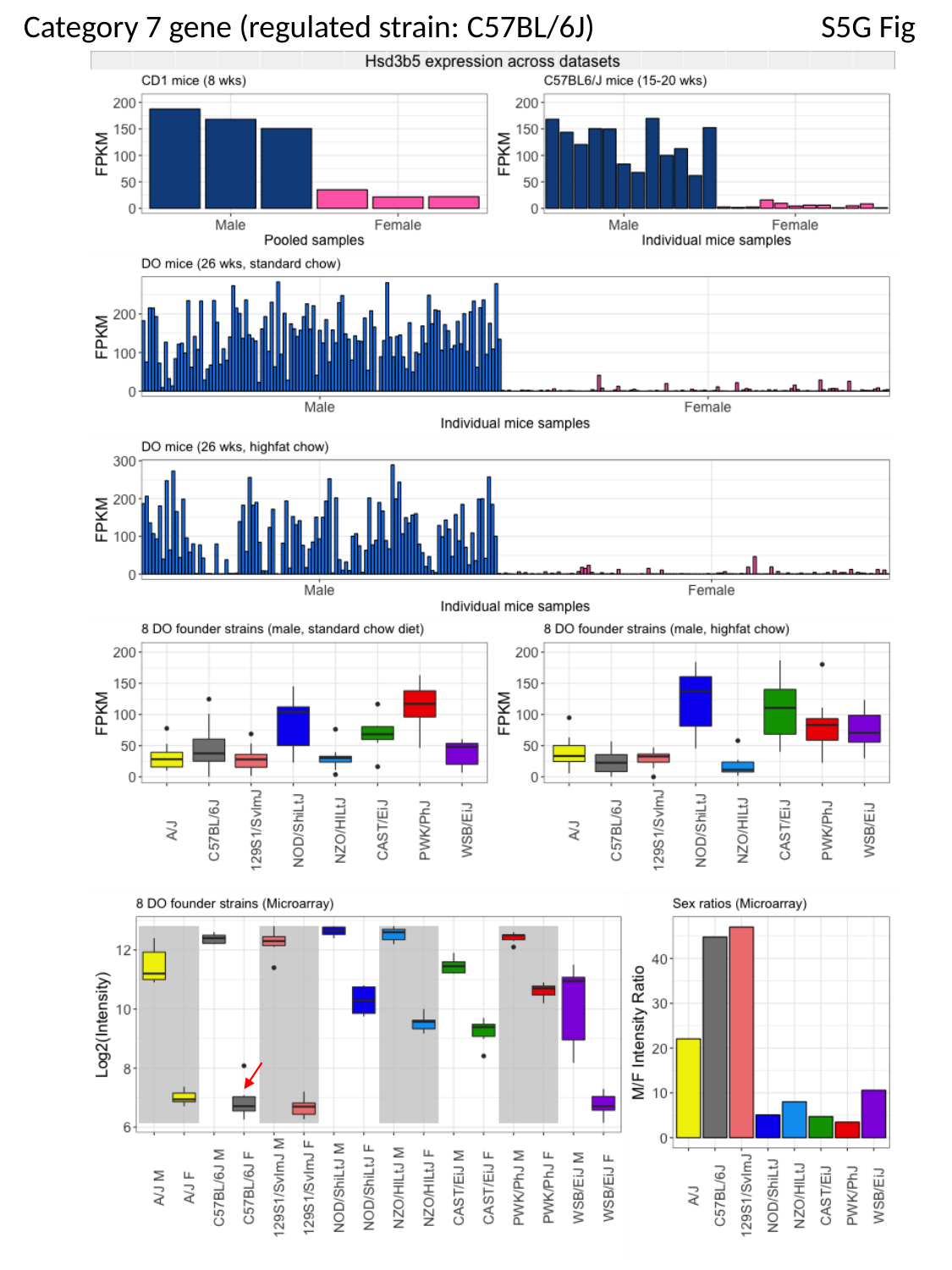

Category 7 gene (regulated strain: C57BL/6J)
S5G Fig

## Slide 14
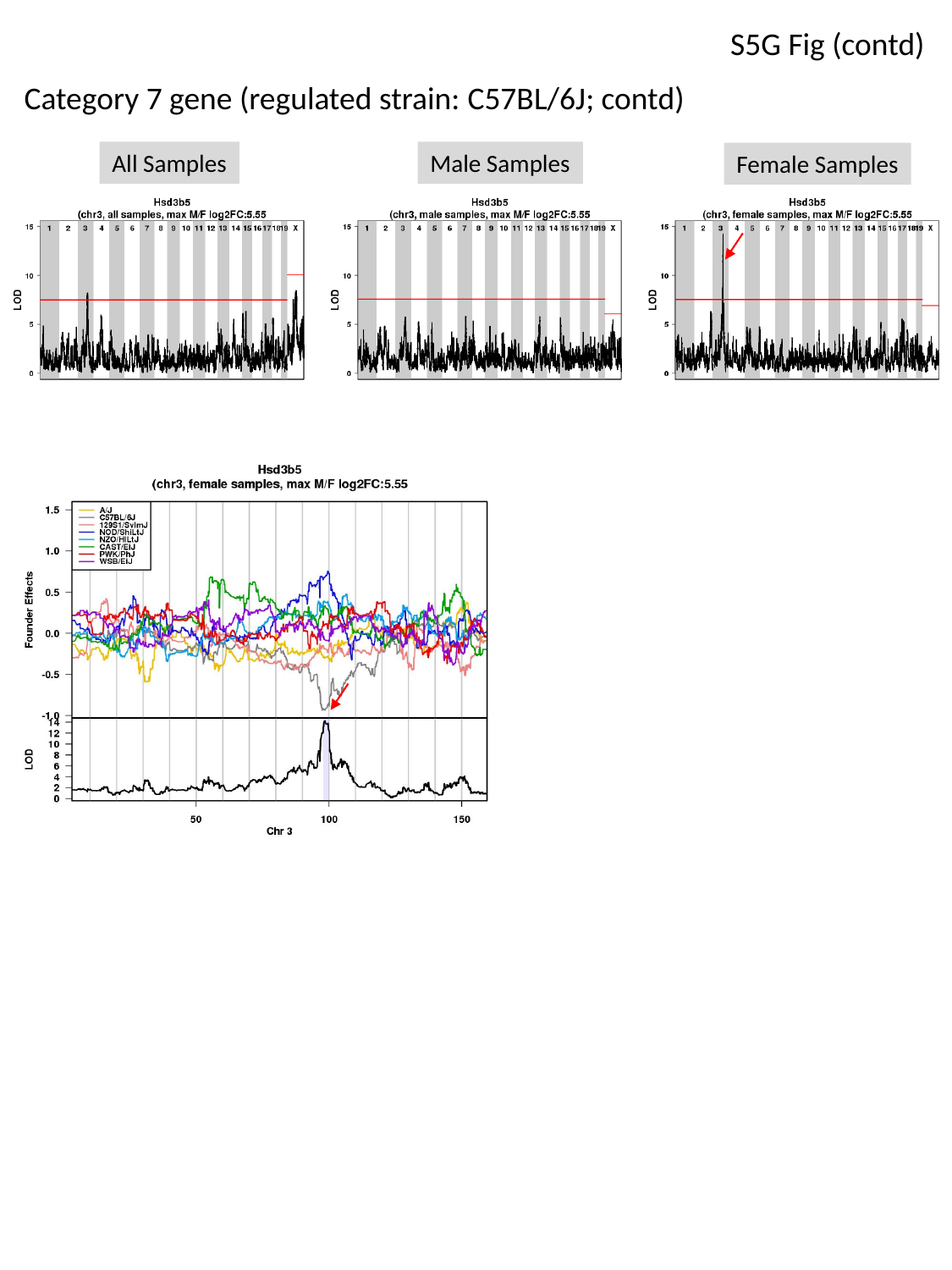

S5G Fig (contd)
Category 7 gene (regulated strain: C57BL/6J; contd)
All Samples
Male Samples
Female Samples

## Slide 15
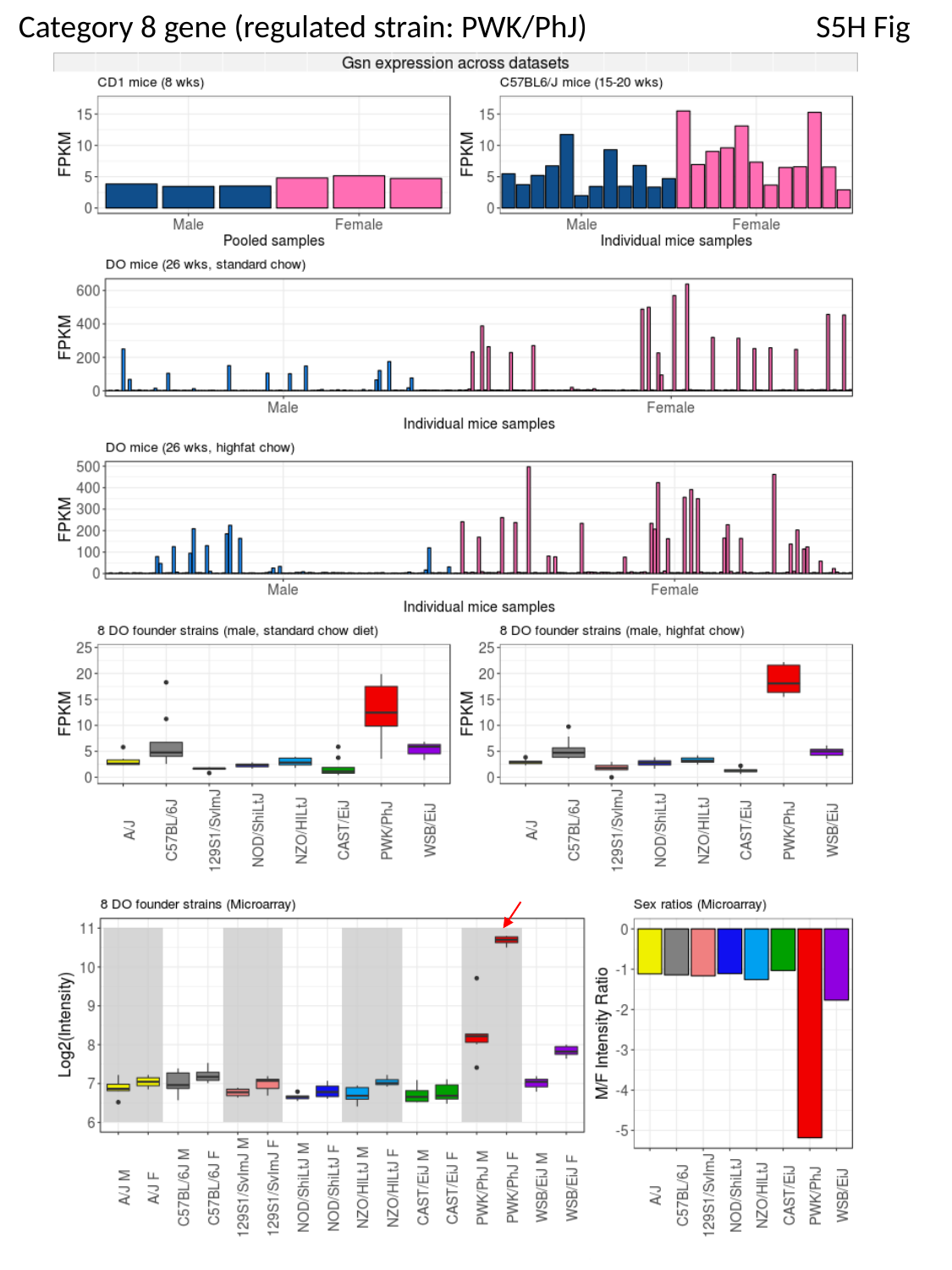

Category 8 gene (regulated strain: PWK/PhJ)
S5H Fig

## Slide 16
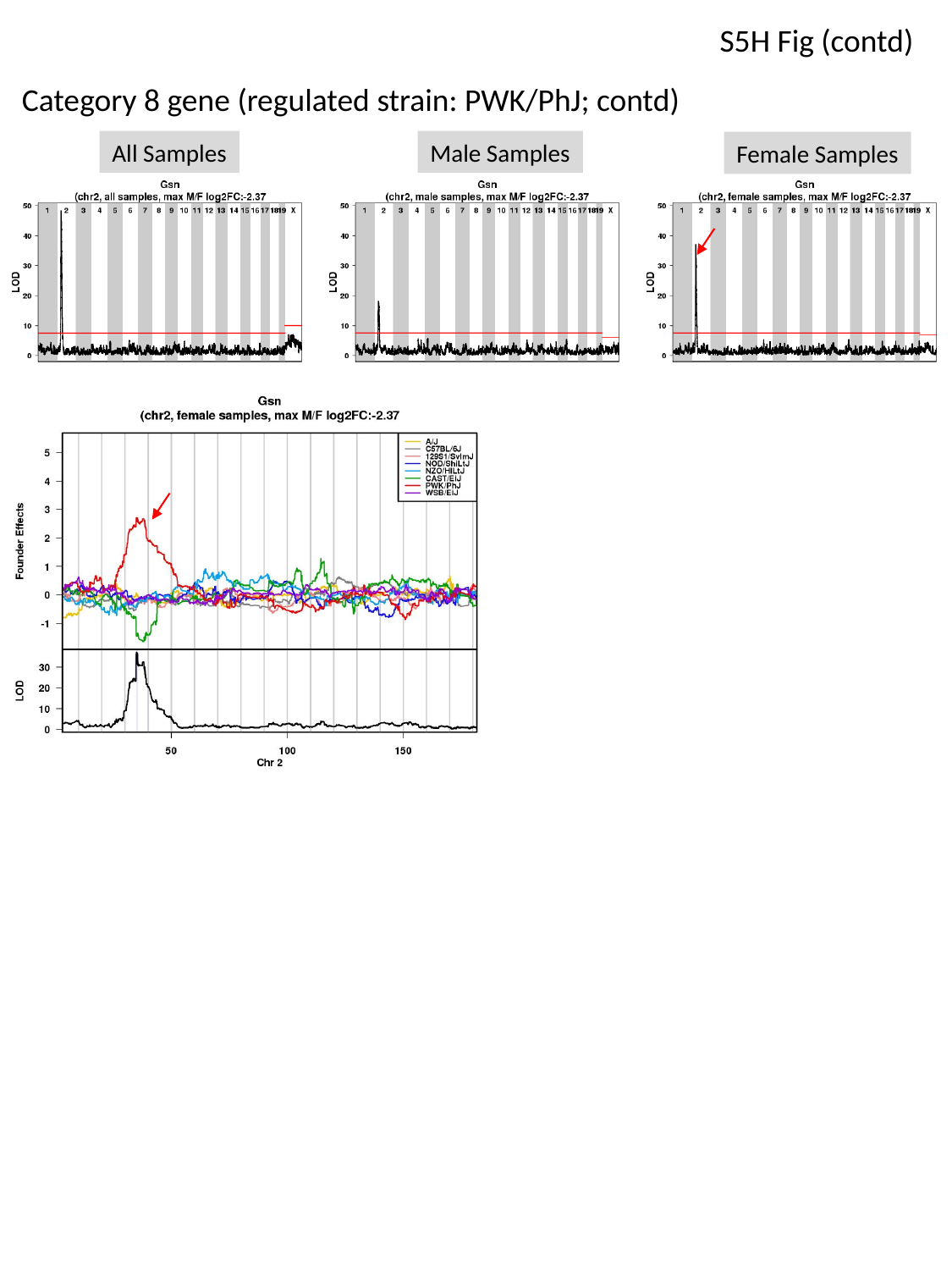

S5H Fig (contd)
Category 8 gene (regulated strain: PWK/PhJ; contd)
All Samples
Male Samples
Female Samples
