# Supplementary material for: Genetic factors contributing to extensive variability of sex-specific hepatic gene expression in Diversity Outbred mice
Source: PLoS One. 2020 Dec 2;15(12):e0242665. doi: 10.1371/journal.pone.0242665 (PMC7710091; doi:10.1371/journal.pone.0242665)
Supplement: S8 Fig — (PPTX) [file pone.0242665.s008.pptx]

## Slide 1
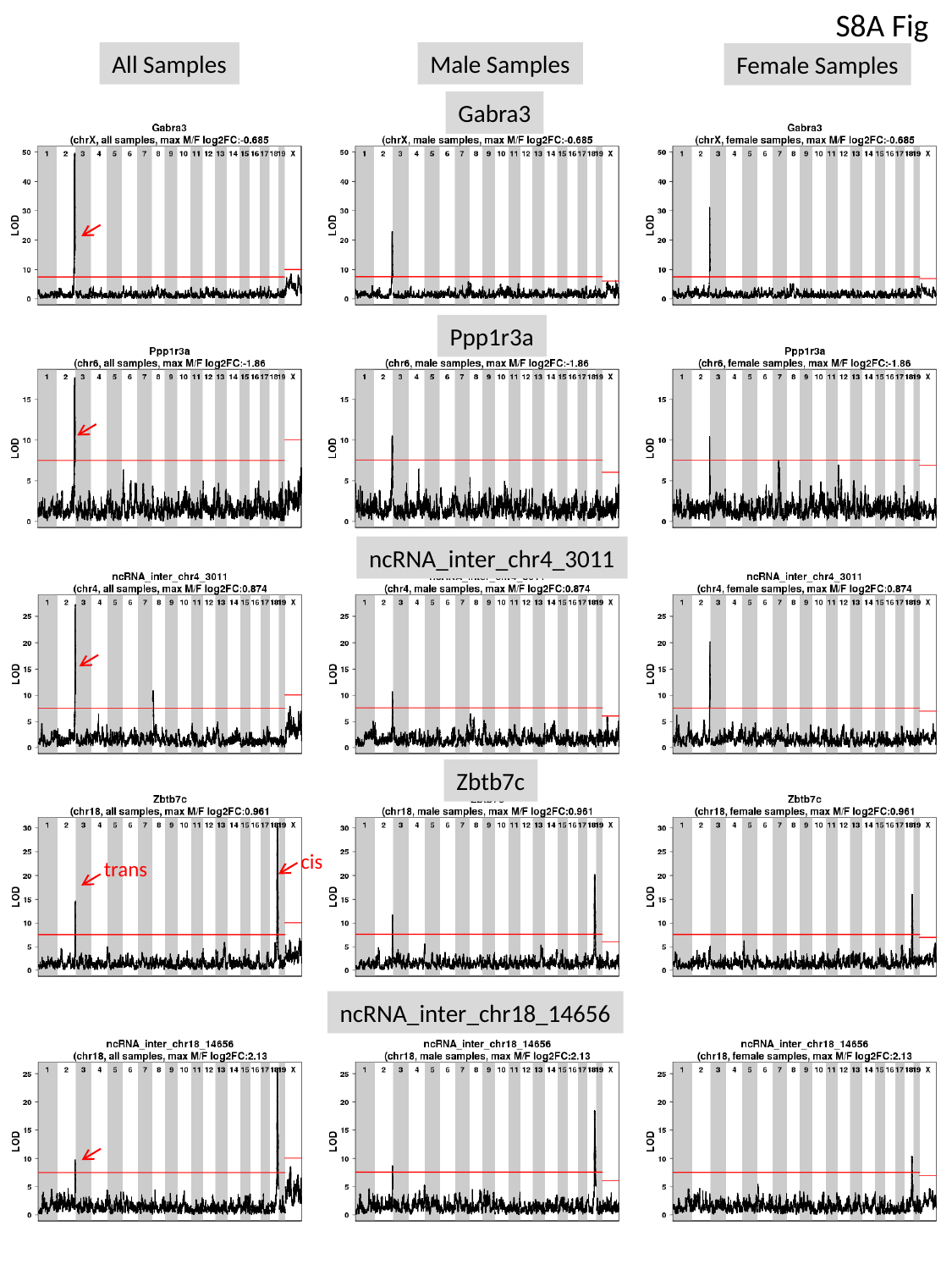

S8A Fig
All Samples
Male Samples
Female Samples
Gabra3
Ppp1r3a
ncRNA_inter_chr4_3011
Zbtb7c
cis
trans
ncRNA_inter_chr18_14656

## Slide 2
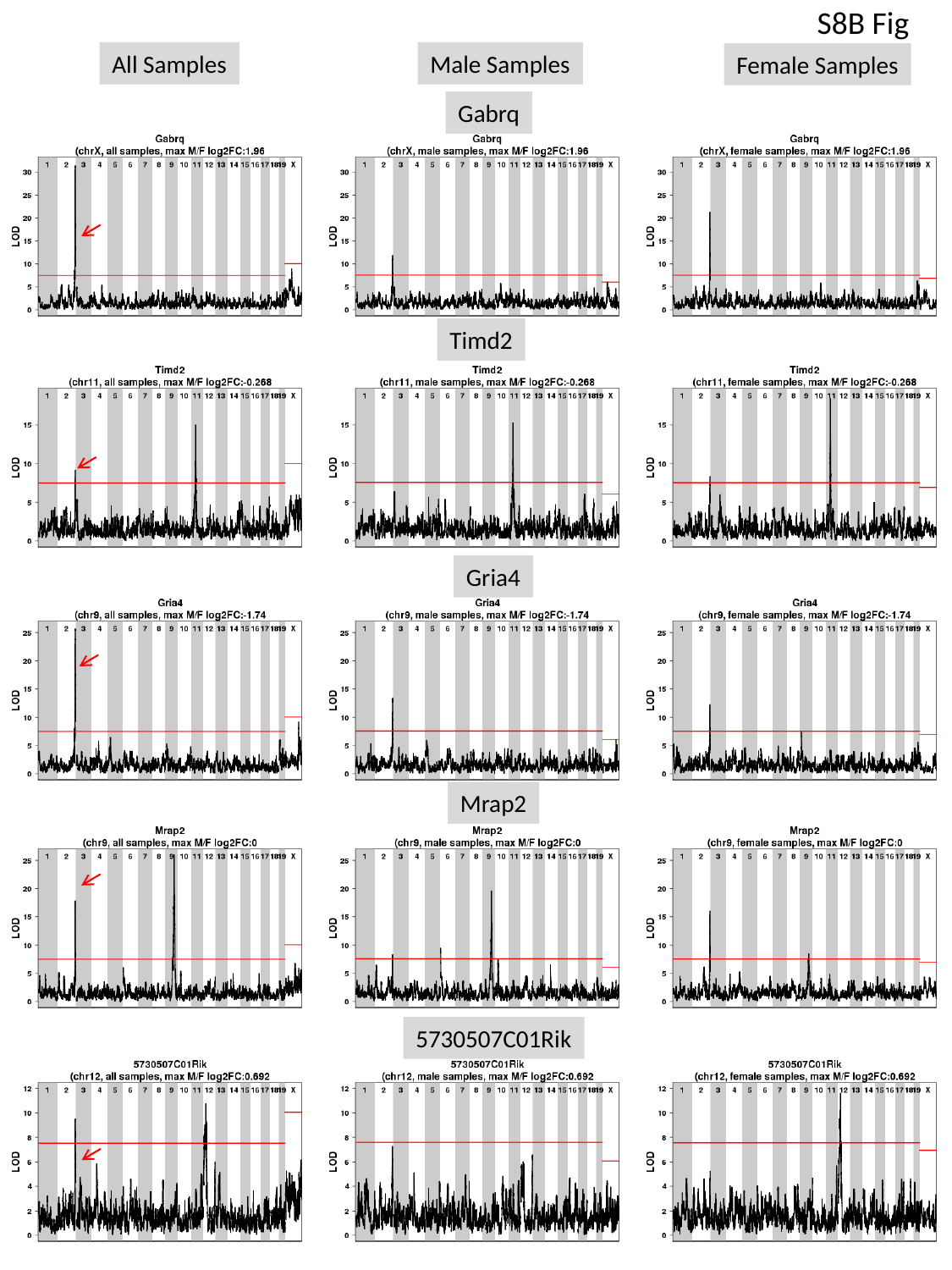

S8B Fig
All Samples
Male Samples
Female Samples
Gabrq
Timd2
Gria4
Mrap2
5730507C01Rik
